# Partial-linear single-index Cox regression models with multiple time-dependent covariates

**DOI:** 10.1186/s12874-024-02434-9

**Published:** 2024-12-20

**Authors:** Myeonggyun Lee, Andrea B. Troxel, Sophia Kwon, George Crowley, Theresa Schwartz, Rachel Zeig-Owens, David J. Prezant, Anna Nolan, Mengling Liu

**Affiliations:** 1 Division of Biostatistics, Department of Population Health, New York University Grossman School of Medicine, 180 Madison Avenue, New York, NY USA; 2 Division of Pulmonary, Critical Care and Sleep Medicine, Department of Medicine, New York University Grossman School of Medicine, New York, NY USA; 3 Bureau of Health Services and Office of Medical Affairs, Fire Department of New York, Brooklyn, NY USA; 4 Department of Epidemiology and Population Health, Albert Einstein College of Medicine, Bronx, NY USA; 5 Pulmonary Medicine Division, Department of Medicine, Montefiore Medical Center and Albert Einstein College of Medicine, Bronx, NY USA

**Keywords:** B-spline smoothing, Semiparametric model, Time-dependent Cox regression, Metabolic syndrome, Lung injury

## Abstract

**Background:**

In cohort studies with time-to-event outcomes, covariates of interest often have values that change over time. The classical Cox regression model can handle time-dependent covariates but assumes linear effects on the log hazard function, which can be limiting in practice. Furthermore, when multiple correlated covariates are studied, it is of great interest to model their joint effects by allowing a flexible functional form and to delineate their relative contributions to survival risk.

**Methods:**

Motivated by the World Trade Center (WTC)-exposed Fire Department of New York cohort study, we proposed a partial-linear single-index Cox (PLSI-Cox) model to investigate the effects of repeatedly measured metabolic syndrome indicators on the risk of developing WTC lung injury associated with particulate matter exposure. The PLSI-Cox model reduces the dimensionality of covariates while providing interpretable estimates of their effects. The model’s flexible link function accommodates nonlinear effects on the log hazard function. We developed an iterative estimation algorithm using spline techniques to model the nonparametric single-index component for potential nonlinear effects, followed by maximum partial likelihood estimation of the parameters.

**Results:**

Extensive simulations showed that the proposed PLSI-Cox model outperformed the classical time-dependent Cox regression model when the true relationship was nonlinear. When the relationship was linear, both the PLSI-Cox model and classical time-dependent Cox regression model performed similarly. In the data application, we found a possible nonlinear joint effect of metabolic syndrome indicators on survival risk. Among the different indicators, BMI had the largest positive effect on the risk of developing lung injury, followed by triglycerides.

**Conclusion:**

The PLSI-Cox models allow for the evaluation of nonlinear effects of covariates and offer insights into their relative importance and direction. These methods provide a powerful set of tools for analyzing data with multiple time-dependent covariates and survival outcomes, potentially offering valuable insights for both current and future studies.

**Supplementary Information:**

The online version contains supplementary material available at 10.1186/s12874-024-02434-9.

## Background

In cohort studies with survival outcomes, multiple covariates of interest may have values that change over time. Time-dependent Cox regression [1] has been widely used to characterize the effects of time-dependent covariates on a time-to-event outcome and is specified as $\:\lambda\:\left(t\right)={\lambda\:}_{0}\left(t\right)\text{exp}\left\{{\beta\:}^{T}X\left(t\right)\right\},$ where $\:{\lambda\:}_{0}\left(t\right)$ is an unknown baseline hazard function and $\:\beta\:$ is a vector of regression coefficients (i.e., log hazards ratio) corresponding to the effects of time-varying covariates $\:X\left(t\right)$. A strong assumption of the Cox regression model is that the covariates $\:X\left(t\right)$ have a linear effect on the log hazard function, which is not always guaranteed in practice. Furthermore, the covariates can be inter-correlated and exhibit complex interactions. Consequently, classical methods may suffer from multicollinearity and inefficient estimates. When multiple time-dependent covariates are studied, we often wish to model their joint effects by allowing a flexible functional form.

Several approaches have been proposed to handle the challenges presented by multiple time-independent covariates. To relax the assumption of linear effects on the log hazard function, nonparametric models [2–6] have been developed to estimate the log hazard function using $\:\lambda\:\left(t\right)={\lambda\:}_{0}\left(t\right)\text{exp}\left\{\psi\:\left(X\right)\right\},$ where $\:\psi\:\left(X\right)$ is an unspecified smooth function of $\:X$. However, unstructured nonparametric function estimation is challenging in practice due to the complexities of high-dimensional data (i.e., curse of dimensionality). To allow for flexibility, semi-structured models have been proposed [7–12]. For example, single-index models [11, 13] have been proposed as $\:\lambda\:\left(t\right)={\lambda\:}_{0}\left(t\right)\text{exp}\left\{\psi\:\left({\beta\:}^{T}X\right)\right\}$, where $\:\psi\:(\bullet\:)$ is an unknown smooth link function consisting of a single index $\:{\beta\:}^{T}X$. Wang [11] proposed the proportional hazards regression models with unknown link function for possible time-dependent covariates, that is, $\:\lambda\:\left(t\right)={\lambda\:}_{0}\left(t\right)\psi\:\left\{{\beta\:}^{T}X\left(t\right)\right\}$. Various techniques such as spline or kernel techniques can be used to approximate the unknown link functions.

Sometimes, major risk factors of interest exhibit nonlinear effects and inter-correlation, while other confounders such as demographics, anthropometric measures, and socioeconomic status can be modeled by linear effects in the proportional hazards model. Sun et al. [14] proposed a partial-linear single-index (PLSI) hazards model to extend the single index model, with the form $\:\lambda\:\left(t\right)={\lambda\:}_{0}\left(t\right)\text{exp}\left\{\psi\:\left({\beta\:}^{T}X\right)+{\alpha\:}^{T}Z\right\},$ in which a set of covariates $\:X$ is modeled using the single index component, while other covariates $\:Z$ maintain their linear form. Even though PLSI models have been widely developed for continuous, binary, ordinal, count, and survival outcomes [15], limited analytical methods currently exist for applying the PLSI survival model to time-varying covariates.

Motivated by our recently published study in the World Trade Center particulate matter-exposed Fire Department of New York (WTC-FDNY) cohort [16] that investigated the time to onset of lung injury after particulate matter exposure, this paper proposed a partial-linear single-index Cox (PLSI-Cox) model with time-dependent covariates. Our scientific question was to assess the totality of overall effects of all five components of metabolic syndrome (MetSyn) – including body mass index (BMI), triglycerides, high density lipoprotein (HDL), glucose, and blood pressure – on the risk of developing WTC-related lung injury (WTC-LI) and to examine their relative importance to inform clinical interventions. Several features of this dataset motivated us to consider the PLSI-Cox model: (1) multiple time-dependent MetSyn covariates are inter-correlated; (2) baseline confounders such as age, race, and smoking status need to be adjusted as linear effects; and (3) the possible nonlinear joint effects of MetSyn components and their relative importance for future intervention. We adopted a B-spline smoothing technique to approximate the unknown link function for the joint effects and used the maximum partial likelihood estimation method for parameter estimates. We also studied asymptotic consistency and normality of the proposed model, which are available in the web appendix.

Our current manuscript is organized as follows. In Sect. 2, we present the model specification, estimation, inference, and implementation of the proposed method. Section 3 includes simulation studies evaluating the finite sample performance of our proposed method. The analysis of the cohort study [16] using our proposed PLSI-Cox model is illustrated in Sect. 4. We conclude in Sect. 5 with discussions and suggestions for further study. Technical details are provided in the web appendix.

## Methods

### Time-dependent PLSI-Cox regression model

Suppose we have an $\:i.i.d.$ sample $\:\left\{{T}_{i},\:{{\Delta\:}}_{i},\:{X}_{i}\left(t\right),\:{Z}_{i}\left(t\right)\right\}$ with $\:n$ subjects $\:\left(i=1,\dots\:,n\right)$. For subject $\:i$, suppose we observe an event time $\:{T}_{i}=\text{min}\left({T}_{i}^{*},{C}_{i}\right)$, where $\:{T}_{i}^{*}$ is the true survival time and $\:{C}_{i}$ is the censoring time, respectively, and a censoring indicator $\:{{\Delta\:}}_{i}=I\left({T}_{i}^{*}\le\:{C}_{i}\right)$, where $\:I(\bullet\:)$ is the indicator function. We assume an independent right censoring scheme in which censoring times are independent of true survival time given the covariate variables. $\:{X}_{i}\left(t\right)$ and $\:{Z}_{i}\left(t\right)$ are respectively $\:p$- and $\:q$-dimensional vectors at time $\:t;t\in\:[0,{\uptau\:}]$. We assume that $\:{X}_{i}\left(t\right)$ includes all possible nonlinear covariates, while $\:{Z}_{i}\left(t\right)$ includes covariates with linear effects and is pre-specified based on prior knowledge (e.g., risk factors for $\:X$ and confounders for $\:Z$). The PLSI-Cox regression model is specified as

1 $$\:{\lambda\:}_{i}\left(t|{X}_{i}\left(t\right),\:{Z}_{i}\left(t\right)\right)={\lambda\:}_{0}\left(t\right)\text{exp}\left\{\psi\:\left({\beta\:}^{T}{X}_{i}\left(t\right)\right)+{\alpha\:}^{T}{Z}_{i}\left(t\right)\right\},$$

where $\:\psi\:\left(\bullet\:\right)$ is the unknown smooth link function, $\:\beta\:$ contains the $\:p$-dimensional contribution weights of $\:X\left(t\right)$, $\:\alpha\:$ is the $\:q$-dimensional regression coefficient vector for $\:Z\left(t\right)$, and $\:{\lambda\:}_{0}\left(t\right)$ is unspecified baseline hazard function. Because $\:\psi\:\left(\bullet\:\right)$ is an unspecified, the relative risk function of $\:{\beta\:}^{T}X\left(t\right)$ can accommodate many flexible forms. To ensure model identifiability, we assume that $\:\psi\:\left(0\right)=0,\:||\beta\:||={\left({\beta\:}^{T}\beta\:\right)}^{1/2}=1$ and the first component of $\:\beta\:$ is positive (i.e., $\:{\beta\:}_{1}>0$). For the implementation, we select one variable with a strong and positive signal as the first component based on prior knowledge, which is a mild condition. Note that the details of these constraints are well described in Sect. 2.1 of Huang and Liu [13].

For the interpretations of our proposed PLSI-Cox model, the regression coefficients $\:\alpha\:$ for $\:Z\left(t\right)$ can be interpreted as usual log hazard ratios. However, due to the presence of the unknown link function, the contribution weights $\:\beta\:$ do not have the usual interpretation as in the standard Cox model. Because we assumed the constraint $\:||\beta\:||=1$, the scale of $\:\beta\:$ represents their relative importance between $\:X\left(t\right)$ while the other terms, $\:\psi\:\left(\bullet\:\right)$ and other variables, are held constant. Moreover, if $\:\psi\:\left(\bullet\:\right)$ is a monotone increasing function, a positive $\:\beta\:$ indicates a higher risk as the covariate value increases, while a negative coefficient suggests a lower risk. Note that Huang and Liu [13] used average derivatives to interpret single-index model when the link function is nonmonotone. Such covariate effects can often be better understood using graphical tools.

### Estimation of our proposed PLSI-Cox model

From our proposed model (1), the partial likelihood function can be constructed as

2 $$\:PL\left(\beta\:,\:\alpha\:,\:\psi\:\right)={{\prod\:}_{i=1}^{n}\left[\frac{\text{exp}\left\{\psi\:\left({\beta\:}^{T}{X}_{i}\left({T}_{i}\right)\right)+{\alpha\:}^{T}{Z}_{i}\left({T}_{i}\right)\right\}}{{\sum\:}_{j=1}^{n}{Y}_{j}\left({T}_{i}\right)\text{exp}\left\{\psi\:\left({\beta\:}^{T}{X}_{j}\left({T}_{i}\right)\right)+{\alpha\:}^{T}{Z}_{j}\left({T}_{i}\right)\right\}}\right]}^{{{\Delta\:}}_{i}},$$

where $\:{Y}_{j}\left(t\right)=I\left({T}_{j}\ge\:t\right)$ indicates the risk set at event time $\:t,\:j=1,\dots\:,n$. In this study, we employ a B-spline basis function to approximate the derivative of the nonlinear component $\:{\psi\:}^{{\prime\:}}\left(\bullet\:\right)$. The B-spline is selected for its numerical stability for implementation, although other basis techniques can be used in principle.

Let $\:{B}_{k}\left(k=1,\dots\:,K\right)$ be the B-spline basis functions with the number of knots $\:K$ [17, 18]. For any $\:\beta\:$ in the neighborhood of its true parameter value, we assume that the support of $\:{\beta\:}^{T}X\left(t\right)$ is a continuous interval $\:\left[c,d\right]$ and $\:0\in\:\left[c,d\right]$, where $\:-\infty\:<c<d<\infty\:$, so that the range of the B-splines can be well defined. In our data application, we standardized $\:X\left(t\right)$ first and found that the algorithm was stable in handling the data range of the single-index. However, in practice we suggest using techniques such as logit transformation or the cumulative distribution function transformation of $\:{\beta\:}^{T}X\left(t\right)$ to convert it into a specific range before applying the B-spline. Thus, we have an approximation represented by

$$\:{\psi\:}^{{\prime\:}}\left({\beta\:}^{T}X\left(t\right)\right)={\sum\:}_{k=1}^{K}{\gamma\:}_{k}{B}_{k}\left({\beta\:}^{T}X\left(t\right)\right)={\gamma\:}^{T}\varvec{B}\left({\beta\:}^{T}X\left(t\right)\right),$$

where $\:{\gamma\:}^{T}={\left({\gamma\:}_{1},\dots\:,\:{\gamma\:}_{K}\right)}^{T}$ and $\:\varvec{B}\left(\mu\:\right)={\left({B}_{1}\left(\mu\:\right),\:\dots\:,\:{B}_{K}\left(\mu\:\right)\right)}^{T}$ as their collection. With the identifiability constraint $\:\psi\:\left(0\right)=0$, we then obtain $\:\psi\:\left({\beta\:}^{T}X\left(t\right)\right)={\gamma\:}^{T}\stackrel{\sim}{\varvec{B}}\left({\beta\:}^{T}X\left(t\right)\right)$, where $\:{\stackrel{\sim}{B}}_{k}\left(\mu\:\right)={\int\:}_{\text{min}\left(0,\mu\:\right)}^{\text{max}\left(0,\:\mu\:\right)}{B}_{k}\left(s\right)ds,\:k=1,\dots\:,K$, are the integrals of the B-spline basis functions, and $\:\stackrel{\sim}{\varvec{B}}\left(\mu\:\right)={\left({\stackrel{\sim}{B}}_{1}\left(\mu\:\right),\:\dots\:,\:{\stackrel{\sim}{B}}_{K}\left(\mu\:\right)\right)}^{T}$. In practice, one typically uses quadratic B-splines in the basis expansion of $\:{\psi\:}^{{\prime\:}}\left(\bullet\:\right)$ so that $\:\psi\:\left(\bullet\:\right)$ is a cubic spline.

Using the B-spline approximation of the unknown link function, the partial likelihood in (2) can be re-written as

$$\:PL\left(\theta\:\right)={{\prod\:}_{i=1}^{n}\left[\frac{\text{exp}\left\{{\gamma\:}^{T}\stackrel{\sim}{\varvec{B}}\left({\beta\:}^{T}{X}_{i}\left({T}_{i}\right)\right)+{\alpha\:}^{T}{Z}_{i}\left({T}_{i}\right)\right\}}{{\sum\:}_{j=1}^{n}{Y}_{j}\left({T}_{i}\right)\text{exp}\left\{{\gamma\:}^{T}\stackrel{\sim}{\varvec{B}}\left({\beta\:}^{T}{X}_{j}\left({T}_{i}\right)\right)+{\alpha\:}^{T}{Z}_{j}\left({T}_{i}\right)\right\}}\right]}^{{{\Delta\:}}_{i}},$$

with the parameter set $\:\theta\:={\left(\gamma\:,\:\beta\:,\:\alpha\:\right)}^{T}$. Based on the construction of the log-partial likelihood function denoted by $\:l\left(\theta\:\right)$, the derivations of the joint score function $\:{S}_{\left(\gamma\:,\:\beta\:,\:\alpha\:\right)}$ of $\:\left(\gamma\:,\:\beta\:,\:\alpha\:\right)$ and the Hessian matrix $\:{H}_{\left(\gamma\:,\:\beta\:,\:\alpha\:\right)}$ are given in Web Appendix A. The log-partial likelihood function $\:l\left(\theta\:\right)$ is a concave function of $\:\left(\gamma\:,\:\alpha\:\right)$ for fixed $\:\beta\:$ because $\:{H}_{\left(\gamma\:,\:\alpha\:\right)}$ is negative semi-definite [14]. Therefore, given fixed $\:\beta\:$, the values of $\:\left(\gamma\:,\:\alpha\:\right)$ that maximize the $\:l\left(\theta\:\right)$ are uniquely defined, if they exist.

For implementation, we develop an iterative estimating procedure:

- **Step 0**. Start with initial values of $\:\alpha\:$ and $\:\beta\:$. For example, the initial values can be obtained from standard time-dependent Cox regression models using R package “survival” with a prespecified $\:\psi\:\left(\bullet\:\right)$ unknown link function which assumes linear coefficients for all covariates.
- **Step 1**. Given the current value of $\:{\widehat{\beta\:}}^{\left(d\right)}$, update the estimates of $\:\gamma\:$ and $\:\alpha\:$ by maximizing the partial likelihood function as

$$PL\left(\gamma,\alpha;{\widehat{\beta}}^{\left(d\right)}\right)={\prod}_{i=1}^{n}{\left[\frac{\text{exp}\left\{\gamma^{T}\stackrel{\sim}{\varvec{B}}\left(\hat{\beta}^{(d)^T}X_i(Ti)\right)+\alpha^{T}Z_i(T_i)\right\}}{{\sum}_{j=1}^{n}{Y}_{j}(T_i)\text{exp}\left\{\gamma^T\stackrel{\sim}{\varvec{B}}\left(\hat{\beta}^{(d)^T}X_j(Ti)\right)\alpha^{T}Z_j(T_i) \right\}}\right]}^{{{\Delta}}_{i}}.$$

In practice, we can perform a classical time-dependent Cox regression model using the covariates of $\stackrel{\sim}{\varvec{B}}\left(\hat{\beta}^{(d)^T}X_i(Ti)\right)$ and $\:{Z}_{i}\left({T}_{i}\right)$ with respect to $\:\gamma\:$ and $\:\alpha\:$, respectively (e.g., coxph() function of “survival” package [19] in R).

- **Step 2**. Given the current values of $\:{\widehat{\gamma\:}}^{(d+1)}$ and $\:{\widehat{\alpha\:}}^{(d+1)}$ from Step 1, update the estimate of $\:\beta\:$ by maximizing the partial likelihood function,

$$\:PL\left(\beta\:;\:{\widehat{\gamma\:}}^{(d+1)},{\widehat{\alpha\:}}^{(d+1)}\right)={\prod\:}_{i=1}^{n}{\left[\frac{{exp}\left({\widehat{\gamma}}^{\left(d+1\right)^T}\stackrel{\sim}{\varvec{B}}\left({\beta\:}^{T}{X}_{i}\left({T}_{i}\right)\right)+{\widehat{\alpha}}^{\left(d+1\right)^T}{Z}_{i}\left({T}_{i}\right)\right)}{{\sum\:}_{j=1}^{n}{Y}_{j}\left({T}_{i}\right){\:exp}\left({\widehat{\gamma}}^{\left(d+1\right)^T}\stackrel{\sim}{\varvec{B}}\left({\beta\:}^{T}{X}_{j}\left({T}_{i}\right)\right)+{\widehat{\alpha}}^{\left(d+1\right)^T}{Z}_{j}\left({T}_{i}\right)\right)}\right]}^{{{\Delta\:}}_{i}}.$$

Then we standardize $\:{\widehat{\beta\:}}^{(d+1)}$ such that $\:||{\widehat{\beta\:}}^{(d+1)}||=1$ and its first component is positive.

- **Step 3**. Repeat Steps 1 and 2 until the parameter convergence criterion is met. In this study, we defined the convergence criterion as $\:\text{max}\left\{\left|{\theta\:}^{new}-{\theta\:}^{old}\right|\right\}<0.0001$.

*Remark 1*. To use existing R packages for Step 2, we employ the Taylor expansion of $\psi\left(\beta^TX\left(t\right)\right)$ at constant $a\left(t\right)={\widehat{\beta}}^{\left(d\right)^T}X\left(t\right)$, that is, $\psi\left(\beta^TX\left(t\right)\right)\approx\psi\left(a\left(t\right)\right)+\left(\beta^TX\left(t\right)-a\left(t\right)\right)\times\psi'\left(a\left(t\right)\right)= {\widehat{\gamma}}^{\left(d+1\right)^T} \widetilde{\boldsymbol B}\left(a\left(t\right)\right)+\left(\beta^TX\left(t\right)-a\left(t\right)\right)\times{\widehat{\gamma}}^{\left(d+1\right)^T} \boldsymbol B\left(a\left(t\right)\right) = \beta^T\left\{X\left(t\right){\widehat{\gamma}}^{\left(d+1\right)^T}\boldsymbol B\left(a\left(t\right)\right)\right\}+\left\{{\widehat{\gamma}}^{\left(d+1\right)^T}\widetilde{\boldsymbol B}\left(a\left(t\right)\right)-a\left(t\right){\widehat{\gamma}}^{\left(d+1\right)^T}\boldsymbol B\left(a\left(t\right)\right)\right\}$. Then, the partial likelihood function in Step 2 can be re-written as

$$\:PL\left(\beta\:;\:{\widehat{\gamma\:}}^{\left(d+1\right)},{\widehat{\alpha\:}}^{\left(d+1\right)},{\widehat{\beta\:}}^{\left(d\right)}\right)={\prod\:}_{i=1}^{n}{\left[\frac{\text{exp}\left\{{\beta\:}^{T}\left\{{X}_{i}\left({T}_{i}\right){\widehat{\gamma}}^{\left(d+1\right)^T}\varvec{B}\left({a}_{i}\left({T}_{i}\right)\right)\right\}+\left\{{\widehat{\gamma}}^{\left(d+1\right)^T}\stackrel{\sim}{\varvec{B}}\left({a}_{i}\left({T}_{i}\right)\right)-{a}_{i}\left({T}_{i}\right)\cdot{\widehat{\gamma}}^{\left(d+1\right)^T}\varvec{B}\left({a}_{i}\left({T}_{i}\right)\right)\right\}+{\widehat{\alpha}}^{\left(d+1\right)^T}{Z}_{i}\left({T}_{i}\right)\right\}}{{\sum\:}_{j=1}^{n}{Y}_{j}\left({T}_{i}\right)\text{exp}\left\{{\beta\:}^{T}\left\{{X}_{j}\left({T}_{i}\right){\widehat{\gamma}}^{\left(d+1\right)^T}\varvec{B}\left({a}_{j}\left({T}_{i}\right)\right)\right\}+\left\{{\widehat{\gamma}}^{\left(d+1\right)^T}\stackrel{\sim}{\varvec{B}}\left({a}_{j}\left({T}_{i}\right)\right)-{a}_{j}\left({T}_{i}\right)\cdot{\widehat{\gamma}}^{\left(d+1\right)^T}\varvec{B}\left({a}_{j}\left({T}_{i}\right)\right)\right\}+{\widehat{\alpha}}^{\left(d+1\right)^T}{Z}_{j}\left({T}_{i}\right)\right\}}\right]}^{{{\Delta\:}}_{i}},$$

with ${a}_{j}\left({T}_{i}\right)={\widehat{\beta}^{(d)^{T}}}X_{j}\left({T}_{i}\right)$. We use $\:{X}_{i}\left({T}_{i}\right){\widehat{\gamma}}^{\left(d+1\right)^T}\varvec{B}\left({a}_{i}\left({T}_{i}\right)\right)$ as our covariate with respect to $\:\beta\:$ and the remaining terms, $\:\left\{{\widehat{\gamma}}^{\left(d+1\right)^T}\stackrel{\sim}{\varvec{B}}\left({a}_{i}\left({T}_{i}\right)\right)-{a}_{i}\left({T}_{i}\right)\cdot{\widehat{\gamma}}^{\left(d+1\right)^T}\varvec{B}\left({a}_{i}\left({T}_{i}\right)\right)\right\}+{\widehat{\alpha}}^{\left(d+1\right)^T}{Z}_{i}\left({T}_{i}\right)$, are constant with the offset (e.g., coxph() function of “survival” package [19] in R, with offset() option).

*Remark 2*. Even though the log-partial likelihood function is concave in but not guaranteed in, the iterative alternating procedure is numerically stable and computationally simple [13, 14, 20]. In our simulation studies, the proposed algorithm performed well and was easily implemented using standard statistical software, R, with existing packages “survival” and “splines2” [19, 21]. Even though our proposed PLSI-Cox model can be estimated by using the profiling approach, it would not be directly implementable using the existing R packages. The R code for our proposed methods is available at https://github.com/ml5977/plsi_survival_models.

### Statistical inference

We first reparametrize $\:\beta\:=\beta\:\left(\sigma\:\right)={\left({\left(1-{||\sigma\:||}^{2}\right)}^{1/2},\:\:{\sigma\:}_{1},\dots\:,{\sigma\:}_{p-1}\right)}^{T}$with $\:\sigma\:={\left({\sigma\:}_{1},\dots\:,{\sigma\:}_{p-1}\right)}^{T}$such that the constraints $\:||\beta\:||=1$ and $\:{\beta\:}_{1}>0$ hold. Note that such reparameterization is solely for the purpose of developing asymptotic theory. Suppose we define a map $\:G:\left(\:\sigma\:,\:\alpha\:,\:\gamma\:\right)\to\:(\:\beta\:,\alpha\:,\:\gamma\:)$, so that $\:\left(\:\beta\:,\alpha\:,\:\gamma\:\right)=G(\sigma\:,\:\alpha\:,\:\gamma\:)$. By the Delta method, the asymptotic variance-covariance matrix can be estimated by

$$\:{{\Sigma\:}}_{\left(\:\widehat{\beta\:},\:\widehat{\alpha\:},\widehat{\gamma\:}\right)}={G}^{{\prime\:}}\left(\widehat{\sigma\:},\:\widehat{\alpha\:},\widehat{\gamma\:}\right){{\Sigma\:}}_{\left(\:\widehat{\sigma\:},\:\widehat{\alpha\:},\widehat{\gamma\:}\right)}{\left[{G}^{{\prime\:}}\left(\widehat{\sigma\:},\:\widehat{\alpha\:},\widehat{\gamma\:}\right)\right]}^{T}=\left[\begin{array}{c}\frac{{\widehat{\beta\:}}_{2}}{{\widehat{\beta\:}}_{1}},\dots\:,\frac{{\widehat{\beta\:}}_{p}}{{\widehat{\beta\:}}_{1}},\:{0}_{1\times\:\left(q+K\right)}\\\:{I}_{p-1+q+K}\end{array}\right]{\left\{-{H}_{\left(\widehat{\sigma\:},\widehat{\alpha\:},\widehat{\gamma\:}\right)}\right\}}^{-1}{\left[\begin{array}{c}\:\frac{{\widehat{\beta\:}}_{2}}{{\widehat{\beta\:}}_{1}},\dots\:,\frac{{\widehat{\beta\:}}_{p}}{{\widehat{\beta\:}}_{1}},\:{0}_{1\times\:\left(q+K\right)}\\\:{I}_{p-1+q+K}\end{array}\right]}^{T},$$

where $\:{I}_{s}$ denotes the $\:s\times\:s$ identity matrix, $\:{0}_{1\times\:\left(q+K\right)}$ denotes the zero vector with dimension of $\:1\times\:\left(q+K\right)$ respectively, and $\:{H}_{(\:\widehat{\sigma\:},\widehat{\alpha\:},\widehat{\gamma\:})}$ is the Hessian matrix of $\:\left(\widehat{\sigma\:},\widehat{\alpha\:},\widehat{\gamma\:}\right)$. Given the regularity conditions and applying martingale theory to our proposed model with time-dependent covariates, we showed that our estimators are consistent and asymptotically normal using the sandwich formular (see Web Appendix B).

The variability of the estimated single-index function $\:\psi\:(\bullet\:)$ evaluated at a fixed $\:s$ can be estimated as $\:{\sigma\:}_{\widehat{\psi\:}\left(s\right)}^{2}=\stackrel{\sim}{B}{\left(s\right)}^{T}{\sigma\:}_{\widehat{\gamma\:}}^{2}\stackrel{\sim}{B}\left(s\right)$. Thus, an approximate 95% pointwise confidence interval (CI) for $\:\psi\:\left(s\right)$ is given by $\:\widehat{\psi\:}\left(s\right)\pm\:1.96{\left\{{\sigma\:}_{\widehat{\psi\:}\left(s\right)}^{2}\right\}}^{1/2}$. Because the analytic form of the standard error (SE) was difficult to implement directly, we used a bootstrapping method, where we resampled subjects with replacement, for the finite-sample SE estimation to compute 95% CIs of $\:\theta\:$ in our simulation study and data application.

### Testing the linearity of single-index function

When fitting the PLSI model, one question of interest is whether the flexible functional form is necessary (i.e., whether classical Cox regression would suffice to fit the data). To test whether the unknown single-index function is linear, the likelihood ratio (LR) test can be performed because the classical time-dependent Cox regression is nested in our proposed PLSI model. Specifically, the test statistic is defined as $\:LR=-2\left(\text{log}\left\{P{L}_{Cox}\right\}-\text{log}\left\{P{L}_{PLSI}\right\}\right),$ where $\:P{L}_{Cox}$ and $\:P{L}_{PLSI}$ denote the values of the partial likelihood for the fitted time-dependent Cox regression and the fitted PLSI-Cox model, respectively. Under the null hypothesis that the classical Cox model holds, the LR test statistic approximately has a $\:{\chi\:}^{2}$ distribution with $\:m$ degrees of freedom, where $\:m=K+d-2$, with $\:K$ being the number of knots and $\:d$ being the degree of the spline [13].

## Simulation study

### Simulation setting

To evaluate the performance of our proposed method, we conducted extensive simulations under various settings. Under the true PLSI-Cox model, that is, $\:{\lambda\:}_{i}\left(t\right)={\lambda\:}_{0}\left(t\right)\text{exp}\left\{\psi\:\left({\beta\:}^{T}X\left(t\right)\right)+{\alpha\:}^{T}Z\left(t\right)\right\}$, we assumed 8 time-dependent covariates $\:X\left(t\right)$ for nonlinear effects and the covariates $\:Z\left(t\right)$ for linear effects were assumed to be time-invariant (i.e., $\:Z\left(t\right)=Z$ for all $\:t$) based on the model structure from our data application. True parameters were set to $\:\beta\:={\left(1,\:-1,\:1,\:-1,\:\text{1,1},\:-1,\:1\right)}^{T}/\sqrt{8}$ for the norm of 1 and $\:\alpha\:={\left(1,\:-1,\:0.5\right)}^{T}$. Based on the true model specification, we generated the survival time from two scenarios of true link function as follows:

i) Linear: $$\:\psi\:\left(s\right)=s$$;
ii) Log curve (nonlinear): $\:\psi\:\left(s\right)=\text{l}\text{o}\text{g}(1+{s}^{2})$.

Under the linear model, we generated time-dependent covariates from $\:{X}_{p}\left(t\right)={\theta\:}_{0p}+{\theta\:}_{1p}t,\:$ where $\:{\theta\:}_{op}\sim N\left(0,2\right)\;\mathrm{for}\;p=1,\;...,8$, $\:{\theta\:}_{1p}\sim U\left(0,0.1\right)\;\mathrm{for}\;\mathrm p\;=\;1,3,5,6,8$, and $\:{\theta\:}_{1p}\sim U\left(-0.1,0\right)\;\mathrm{for}\;p\mathit\;=\;2,4,7$ and time-independent covariates from $\:Z_1,Z_2\sim N\left(0,2\right)$ and $\:Z_3\sim Bern\left(p=0.5\right)$. The baseline hazard function was $\:{\lambda\:}_{0}\left(t\right)=\text{exp}\left(-2.3\right)$. On the other hand, for the log curve model our covariates were generated from $\:{X}_{p}\left(t\right)={\theta\:}_{o}+{\theta\:}_{1}t,\:$ where $\:{\theta\:}_o\sim N\left(0,1\right)$ and $\:{\theta\:}_1\sim N\left(0,0.05\right)$ for $\:p=1,\dots\:,8$, $\:Z_1,\:Z_2\sim U\left(-0.2,\;0.2\right)$ and $\:Z_3\sim Bern\left(p=0.5\right)$. The baseline hazard function $\:{\lambda\:}_{0}\left(t\right)$ was set to $\:\text{exp}\left(-6.9\right)$.

We considered sample sizes of 500 and 300 and specified 25% and 50% censoring rates using a fixed censoring time at the end of the study. We further investigated the performance of the PLSI-Cox model under smaller sample size, random censoring mechanism and high-correlation setting (see Web Appendix C). The number of repeated observations for time-dependent covariates per subject was generated from a discrete uniform distribution on $\:\left\{1,\dots\:,5\right\}$ including a baseline measurement at time $\:t=0$. The observed measurement time was randomly selected between 0 and the observed survival time for each subject. For simplicity, we used 3 equally spaced knots in the range of $\:{\beta\:}^{T}X\left(t\right)$ for the B-spline approximation and applied a convergence criterion of $\:{10}^{-4}$ for each iteration. Note that the performance was not sensitive to the number of knots in a reasonable range (e.g., one to five knots) under our simulation. For each setting, we ran 500 simulations.

Using the generated dataset, we fitted our proposed PLSI-Cox model and the classical time-dependent Cox regression model. We used 500 bootstrap samples to compute standard errors of estimates. Note that we rescaled the estimates $\:\beta\:$ of the classical time-dependent Cox regression such that the coefficient vector had the same norm of 1 as for the proposed PLSI model. To evaluate the estimated coefficients for $\:\theta\:={\left(\gamma\:,\:\beta\:,\:\alpha\:\right)}^{T}$, we reported performance measures: (1) Bias: the average of $\:\left\{\widehat{\theta\:}-\theta\:\right\}$, (2) SD: the sample standard deviation of $\:\widehat{\theta\:}$, (3) SE: the average of estimated standard errors of $\:\theta\:$ by 500 bootstrap samples, and (4) CP: the coverage probability of the 95% CI for $\:\theta\:$. For the estimated link function, we reported the mean of the estimated single-index function $\:\widehat{\psi\:}(\bullet\:)$ and 95% CIs, constructed using the 2.5% and 97.5% sample quantiles of the estimated link function from 500 simulations. The rate of convergence (i.e., percent converged out of 500 simulations) was also reported.

Using the LR test statistic, we examined the type I error and power of the proposed method. To investigate the change in power, $\:\psi\:\left(s\right)=\text{log}\left(1+{s}^{2}I(s\ge\:\eta\:)\right)$, where $\:\eta\:=0,\:-1\:$and $\:-\infty\:$ (i.e., $\:s\ge\:\eta\:$), and $\:\eta\:=-\infty\:$ indicates no truncation and the nonlinear relationship becomes severe when the truncated value $\:\eta\:$ goes to $\:-\infty\:$. Note that an additional 500 simulations with sample sizes of 200 and 300 were conducted under 10% and 25% censoring rates to compute type I error and power. All computations were performed using R software (version 4.1.2).

### Simulation results

Table 1 showed the results of the linear model and indicates that both the proposed method and classical time-dependent Cox model estimate the parameters reasonably well. Under the linear setting, the performance of the time-dependent Cox model was considered the gold standard, indicating empirically unbiased and reasonably efficient results. Our proposed PLSI-Cox model showed proper results with the empirical coverage probabilities (CPs) of the 95% CIs for $\:\beta\:$ and $\:\alpha\:$ close to the nominal level. The biases of parameter estimations were small, and standard deviations (SDs) of the estimates were close to the empirical standard errors (SEs). Compared to the results of the gold standard when the link function was linear, PLSI-Cox yielded a slightly larger SEs but maintain good efficiency. When sample size increased, both the biases and standard errors of estimates of $\:\beta\:$ and $\:\alpha\:$ tended to decrease, which is not surprising.

**Table 1:** Linear model: simulation results of parameter estimations

	Time-dependent Cox model				Proposed PLSI-Cox model			
	Bias	SD	SE	CP	Bias	SD	SE	CP
*N* = 500 with censoring rate 25% (100% converged)								
$\:{\beta\:}_{1}$	−0.001	0.036	0.035	0.938	−0.001	0.037	0.036	0.952
$\:{\beta\:}_{2}$	0.003	0.035	0.035	0.950	0.003	0.035	0.037	0.956
$\:{\beta\:}_{3}$	−0.001	0.036	0.035	0.942	−0.001	0.037	0.037	0.948
$\:{\beta\:}_{4}$	0.002	0.036	0.035	0.948	0.002	0.036	0.037	0.952
$\:{\beta\:}_{5}$	−0.001	0.036	0.035	0.954	−0.002	0.037	0.036	0.958
$\:{\beta\:}_{6}$	−0.002	0.035	0.035	0.942	−0.002	0.036	0.037	0.940
$\:{\beta\:}_{7}$	0.001	0.036	0.035	0.936	0.001	0.036	0.036	0.942
$\:{\beta\:}_{8}$	−0.004	0.037	0.036	0.946	−0.004	0.037	0.037	0.958
$\:{\alpha\:}_{1}$	0.000	0.055	0.055	0.944	0.001	0.056	0.056	0.944
$\:{\alpha\:}_{2}$	0.000	0.053	0.056	0.964	−0.003	0.053	0.056	0.972
$\:{\alpha\:}_{3}$	0.002	0.114	0.111	0.936	0.002	0.114	0.113	0.938
*N* = 500 with censoring rate 50% (100% converged)								
$\:{\beta\:}_{1}$	−0.002	0.041	0.043	0.954	−0.002	0.042	0.045	0.954
$\:{\beta\:}_{2}$	0.005	0.043	0.043	0.940	0.005	0.045	0.045	0.940
$\:{\beta\:}_{3}$	−0.003	0.043	0.043	0.938	−0.004	0.044	0.045	0.948
$\:{\beta\:}_{4}$	0.003	0.044	0.043	0.936	0.003	0.045	0.045	0.938
$\:{\beta\:}_{5}$	−0.005	0.044	0.043	0.948	−0.006	0.045	0.045	0.946
$\:{\beta\:}_{6}$	0.001	0.044	0.043	0.932	0.002	0.045	0.045	0.934
$\:{\beta\:}_{7}$	0.002	0.043	0.043	0.952	0.002	0.044	0.045	0.948
$\:{\beta\:}_{8}$	−0.003	0.042	0.043	0.942	−0.004	0.043	0.045	0.950
$\:{\alpha\:}_{1}$	0.022	0.063	0.068	0.956	0.025	0.063	0.070	0.956
$\:{\alpha\:}_{2}$	−0.023	0.066	0.068	0.956	−0.026	0.067	0.070	0.946
$\:{\alpha\:}_{3}$	0.015	0.138	0.140	0.958	0.017	0.139	0.142	0.956
*N* = 300 with censoring rate 25% (100% converged)								
$\:{\beta\:}_{1}$	−0.002	0.048	0.046	0.954	−0.002	0.049	0.050	0.952
$\:{\beta\:}_{2}$	0.000	0.048	0.047	0.950	0.000	0.049	0.050	0.954
$\:{\beta\:}_{3}$	−0.003	0.047	0.047	0.950	−0.003	0.049	0.050	0.956
$\:{\beta\:}_{4}$	0.005	0.047	0.047	0.934	0.004	0.047	0.050	0.954
$\:{\beta\:}_{5}$	−0.004	0.048	0.047	0.938	−0.005	0.048	0.050	0.946
$\:{\beta\:}_{6}$	−0.002	0.047	0.047	0.950	−0.001	0.047	0.050	0.956
$\:{\beta\:}_{7}$	0.003	0.047	0.047	0.938	0.003	0.048	0.050	0.950
$\:{\beta\:}_{8}$	−0.007	0.047	0.047	0.940	−0.007	0.048	0.051	0.948
$\:{\alpha\:}_{1}$	0.019	0.070	0.077	0.964	0.023	0.071	0.079	0.968
$\:{\alpha\:}_{2}$	−0.015	0.074	0.077	0.952	−0.019	0.075	0.079	0.956
$\:{\alpha\:}_{3}$	0.008	0.144	0.151	0.960	0.013	0.146	0.156	0.956
*N* = 300 with censoring rate 50% (100% converged)								
$\:{\beta\:}_{1}$	−0.007	0.056	0.056	0.948	−0.007	0.058	0.062	0.952
$\:{\beta\:}_{2}$	0.002	0.057	0.057	0.944	0.003	0.059	0.062	0.952
$\:{\beta\:}_{3}$	−0.003	0.057	0.056	0.942	−0.002	0.059	0.062	0.950
$\:{\beta\:}_{4}$	0.006	0.057	0.057	0.946	0.006	0.058	0.062	0.956
$\:{\beta\:}_{5}$	−0.005	0.054	0.057	0.962	−0.005	0.056	0.063	0.972
$\:{\beta\:}_{6}$	−0.006	0.060	0.056	0.938	−0.006	0.063	0.063	0.942
$\:{\beta\:}_{7}$	0.004	0.054	0.057	0.956	0.006	0.055	0.063	0.974
$\:{\beta\:}_{8}$	−0.003	0.058	0.057	0.936	−0.004	0.059	0.062	0.952
$\:{\alpha\:}_{1}$	0.043	0.092	0.096	0.944	0.049	0.094	0.100	0.948
$\:{\alpha\:}_{2}$	−0.040	0.091	0.096	0.950	−0.045	0.093	0.100	0.948
$\:{\alpha\:}_{3}$	−0.001	0.193	0.193	0.950	0.002	0.197	0.202	0.950

On the other hand, when the true link function was nonlinear, the classical Cox model was substantially biased (Table 2). Moreover, the coverage probabilities for the classical method revealed inaccurate estimates of the standard errors, especially for the nonlinear effects $\:\beta\:$. However, our proposed PLSI-Cox model consistently performed well and showed unbiased results in estimation and reasonable values of standard errors (Table 2). The coverage probabilities also were close to the nominal level at 0.95. The results for linear and nonlinear cases were similar with different censoring rates. Thus, the proposed PLSI-Cox model showed efficient estimates when the true link function was both linear and nonlinear.

**Table 2:** Nonlinear model: simulation results of parameter estimations

	Time-dependent Cox model				Proposed PLSI-Cox model			
	Bias	SD	SE	CP	Bias	SD	SE	CP
*N* = 500 with censoring rate 25% (99.8% converged)								
$\:{\beta\:}_{1}$	−0.344	0.350	0.247	0.634	0.000	0.014	0.014	0.946
$\:{\beta\:}_{2}$	0.343	0.338	0.245	0.660	0.000	0.015	0.014	0.938
$\:{\beta\:}_{3}$	−0.359	0.340	0.248	0.638	0.000	0.014	0.014	0.932
$\:{\beta\:}_{4}$	0.357	0.326	0.244	0.636	0.000	0.015	0.014	0.930
$\:{\beta\:}_{5}$	−0.344	0.349	0.246	0.638	−0.002	0.014	0.014	0.948
$\:{\beta\:}_{6}$	−0.370	0.381	0.275	0.650	0.000	0.017	0.017	0.958
$\:{\beta\:}_{7}$	0.360	0.374	0.268	0.670	−0.001	0.016	0.015	0.934
$\:{\beta\:}_{8}$	−0.350	0.369	0.275	0.690	0.000	0.017	0.017	0.952
$\:{\alpha\:}_{1}$	−0.488	0.431	0.431	0.774	−0.067	0.408	0.423	0.954
$\:{\alpha\:}_{2}$	0.455	0.409	0.430	0.848	0.053	0.398	0.425	0.974
$\:{\alpha\:}_{3}$	−0.231	0.097	0.099	0.380	−0.023	0.099	0.099	0.948
*N* = 500 with censoring rate 50% (100% converged)								
$\:{\beta\:}_{1}$	−0.362	0.344	0.250	0.650	0.000	0.014	0.014	0.938
$\:{\beta\:}_{2}$	0.361	0.342	0.249	0.638	0.001	0.015	0.014	0.938
$\:{\beta\:}_{3}$	−0.341	0.327	0.250	0.672	−0.001	0.014	0.014	0.952
$\:{\beta\:}_{4}$	0.379	0.342	0.247	0.632	0.001	0.013	0.014	0.958
$\:{\beta\:}_{5}$	−0.349	0.346	0.251	0.670	−0.001	0.015	0.014	0.940
$\:{\beta\:}_{6}$	−0.330	0.387	0.275	0.678	−0.001	0.017	0.017	0.950
$\:{\beta\:}_{7}$	0.357	0.359	0.270	0.660	0.000	0.016	0.015	0.938
$\:{\beta\:}_{8}$	−0.364	0.378	0.278	0.672	0.002	0.018	0.017	0.938
$\:{\alpha\:}_{1}$	−0.481	0.419	0.430	0.798	−0.089	0.394	0.422	0.962
$\:{\alpha\:}_{2}$	0.471	0.431	0.429	0.814	0.093	0.423	0.422	0.952
$\:{\alpha\:}_{3}$	−0.232	0.092	0.099	0.348	−0.022	0.096	0.099	0.952
*N* = 300 with censoring rate 25% (99.8% converged)								
$\:{\beta\:}_{1}$	−0.321	0.336	0.245	0.688	−0.001	0.018	0.019	0.962
$\:{\beta\:}_{2}$	0.356	0.343	0.243	0.638	0.001	0.017	0.019	0.960
$\:{\beta\:}_{3}$	−0.359	0.340	0.244	0.622	0.000	0.019	0.019	0.952
$\:{\beta\:}_{4}$	0.355	0.350	0.241	0.618	0.001	0.018	0.019	0.968
$\:{\beta\:}_{5}$	−0.347	0.353	0.245	0.628	−0.001	0.019	0.019	0.944
$\:{\beta\:}_{6}$	−0.340	0.372	0.272	0.668	0.000	0.022	0.024	0.966
$\:{\beta\:}_{7}$	0.335	0.367	0.266	0.676	−0.001	0.020	0.021	0.968
$\:{\beta\:}_{8}$	−0.333	0.365	0.274	0.700	0.000	0.021	0.024	0.966
$\:{\alpha\:}_{1}$	−0.447	0.556	0.574	0.882	−0.026	0.572	0.567	0.956
$\:{\alpha\:}_{2}$	0.481	0.564	0.575	0.872	0.094	0.532	0.571	0.968
$\:{\alpha\:}_{3}$	−0.235	0.134	0.132	0.594	−0.026	0.127	0.133	0.954
*N* = 300 with censoring rate 50% (100% converged)								
$\:{\beta\:}_{1}$	−0.401	0.346	0.248	0.600	−0.002	0.019	0.019	0.948
$\:{\beta\:}_{2}$	0.392	0.333	0.247	0.600	0.000	0.018	0.019	0.956
$\:{\beta\:}_{3}$	−0.361	0.346	0.250	0.630	0.000	0.019	0.020	0.958
$\:{\beta\:}_{4}$	0.367	0.342	0.245	0.648	0.002	0.018	0.019	0.954
$\:{\beta\:}_{5}$	−0.379	0.335	0.250	0.618	−0.001	0.019	0.019	0.942
$\:{\beta\:}_{6}$	−0.354	0.381	0.276	0.660	0.000	0.023	0.024	0.962
$\:{\beta\:}_{7}$	0.355	0.360	0.268	0.644	−0.001	0.021	0.021	0.948
$\:{\beta\:}_{8}$	−0.364	0.379	0.277	0.662	−0.001	0.024	0.024	0.936
$\:{\alpha\:}_{1}$	−0.420	0.565	0.572	0.902	−0.011	0.566	0.567	0.936
$\:{\alpha\:}_{2}$	0.440	0.553	0.572	0.878	0.018	0.568	0.566	0.956
$\:{\alpha\:}_{3}$	−0.236	0.130	0.132	0.598	−0.030	0.125	0.131	0.962

**Fig. 1:**
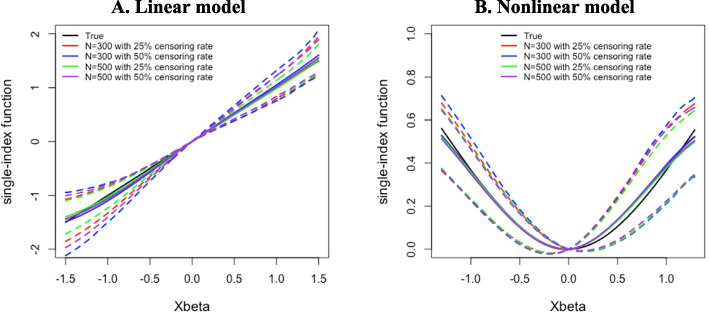
The mean of estimated single-index function with 95% confidence intervals under (**A**) linear single-index function and (**B**) log curve single-index function. Each colored link represents *N* = 300 with a 25% censoring rate (red) and a 50% censoring rate (blue), and *N *= 500 with a 25% censoring rate (green) and a 50% censoring rate (purple). The black solid line represents the true link function [Color figure online]

Figure 1 showed the mean of the estimated function $\:\psi\:(\bullet\:)$ with 95% CIs. Our proposed method demonstrated that the estimated function approximates the true function closely, indicating good performance for both linear and nonlinear link function cases. The proposed method performed well even when the sample size was small or the censoring rate was relatively high (Fig. 1). The results of our simulation study were robust under conditions of smaller sample size, random censoring distribution, and high-correlation settings (Tables S1–S3 in Web Appendix C).

The results of the empirical size and power using the LR test statistic are given in Table 3. Under different sample sizes and censoring rates, the empirical sizes were consistently close to 0.05. Our simulation also demonstrated that the power increased when the nonlinear relationship became severe, which is not surprising. When we used truncated values such as $\:\eta\:=0$, the power was relatively lower in the range from 0.15 to 0.22 because the truncated link function is close to a linear. However, when the log curve model is true with no truncation (i.e., $\:\eta\:=-\infty\:$), the power was 1.00 indicating that the null hypothesis as rejected for each of the 500 simulations. Moreover, the power also increased when the sample size increased as expected.

**Table 3:** Empirical size and power using LR test statistic with 500 simulations

*N*	Censoring Rate	Size^a^	Power^b^		
			$\:\eta\:=0$	$\:\eta\:=-1$	$\:\eta\:=-\infty\:$ ^c^
200	10%	0.056	0.160	0.688	1.000
	25%	0.058	0.151	0.656	1.000
300	10%	0.048	0.220	0.863	1.000
	25%	0.056	0.191	0.792	1.000

^a^Size was calculated under linear true link function $\:\left(\psi\:\left(t\right)=t\right)$

^b^Power was calculated under log true link function $\:(\psi\:\left(t\right)=\text{l}\text{o}\text{g}(1+{t}^{2})$ with truncated $\:t$ at $\:\eta\:$ (i.e. $\:t\ge\:\eta\:$). Note that due to truncated dataset sample size was not exactly same as either 200 or 300. The sample size was very close to the setting

^c^No truncation

## Data application

As stated in our recent manuscript [16], 5,738 participants in the WTC-FDNY cohort were longitudinally followed and underwent serial pulmonary function tests (PFTs), complete blood count, chemistries, and lipids as per our recent publication. The details and results of this cohort study were reported in Kwon et al. [16]. Our clinical outcome of interest was time to first onset of WTC-LI, defined as Forced Expiratory Volume in 1 s (FEV_1_) percent predicted $\:<$ lower limit of normal). Longitudinal MetSyn data of BMI, triglycerides, HDL, glucose, systolic blood pressure (SBP), and diastolic blood pressure (DBP) were assessed [16]. Baseline information and demographics were previously published in Table 1 of Kwon et al. [16]. Cases (*n* = 1,475) did not significantly differ from controls (*n* = 4,263) with respect to baseline age, gender, or race as per prior report. However, cases were more likely to be smokers, have higher WTC-particulate exposure and have different patterns of MetSyn components than controls (Table 1 of Kwon et al. [16]).

We applied our PLSI-Cox model to assess the possibly nonlinear joint effect of MetSyn components and to delineate their relative contributions to the risk of developing lung injury [16]. Due to right-skewness, we log-transformed triglycerides and glucose. After log-transformation, all MetSyn components were standardized to mean 0 and standard deviation of 1 for model stability. We used 3 knots for the B-spline technique.

The estimated parameters, corresponding standard errors by 5,000 bootstrap samples, and p-values are presented in Table 4, adapted from Supplemental Table E5 in Kwon et al. [16]. As in our recent publication, we found that BMI had the largest magnitude and positive weight (0.733) on the risk of developing lung injury after particulate matter exposure, followed by log-transformed triglycerides (0.509) and HDL (−0.418). HDL had negative weight for survival risk, which is clinically reasonable. Among baseline variables, ever-smoking was a significant risk factor with the estimated hazard ratio (HR) of 1.200 ($\:={e}^{0.182}$) [16].

**Table 4:** Results of parameter estimations of the MetSyn cohort study using PLSI-Cox model

	Estimates	SE^a^	*P*-value^a^
**MetSyn component**^b^			
BMI	0.733	0.175	< 0.001
Log (Triglycerides)	0.509	0.201	0.011
HDL	−0.418	0.201	0.038
Log (Glucose)	−0.167	0.147	0.256
SBP	0.011	0.178	0.951
DBP	0.066	0.173	0.702
**Baseline information**			
Race (Caucasian)	0.035	0.118	0.767
Baseline age	−0.005	0.004	0.223
Smoking status (ever)	0.182	0.062	0.003
Exposure (high)	0.176	0.075	0.020

^a^5,000 bootstrap samples were used

^b^All MetSyn components were standardized

Adapted with permission of the American Thoracic Society. Copyright © 2023 American Thoracic Society. All rights reserved. Cite: Kwon S, Lee M, Crowley G, Schwartz T, Zeig-Owens R, Prezant DJ, Liu M, and Nolan A /2021 / Dynamic Metabolic Risk Profiling of World Trade Center Lung Disease: A Longitudinal Cohort Study /Am J Respir Crit Care Med / Vol 204(9) / 1035-1047. The American Journal of Respiratory and Critical Care Medicine is an official journal of the American Thoracic Society. Readers are encouraged to read the entire article for the correct context at [https://www.atsjournals.org/doi/full/10.1164/rccm.202006-2617OC]. The authors, editors, and The American Thoracic Society are not responsible for errors or omissions in adaptations

**Fig. 2:**
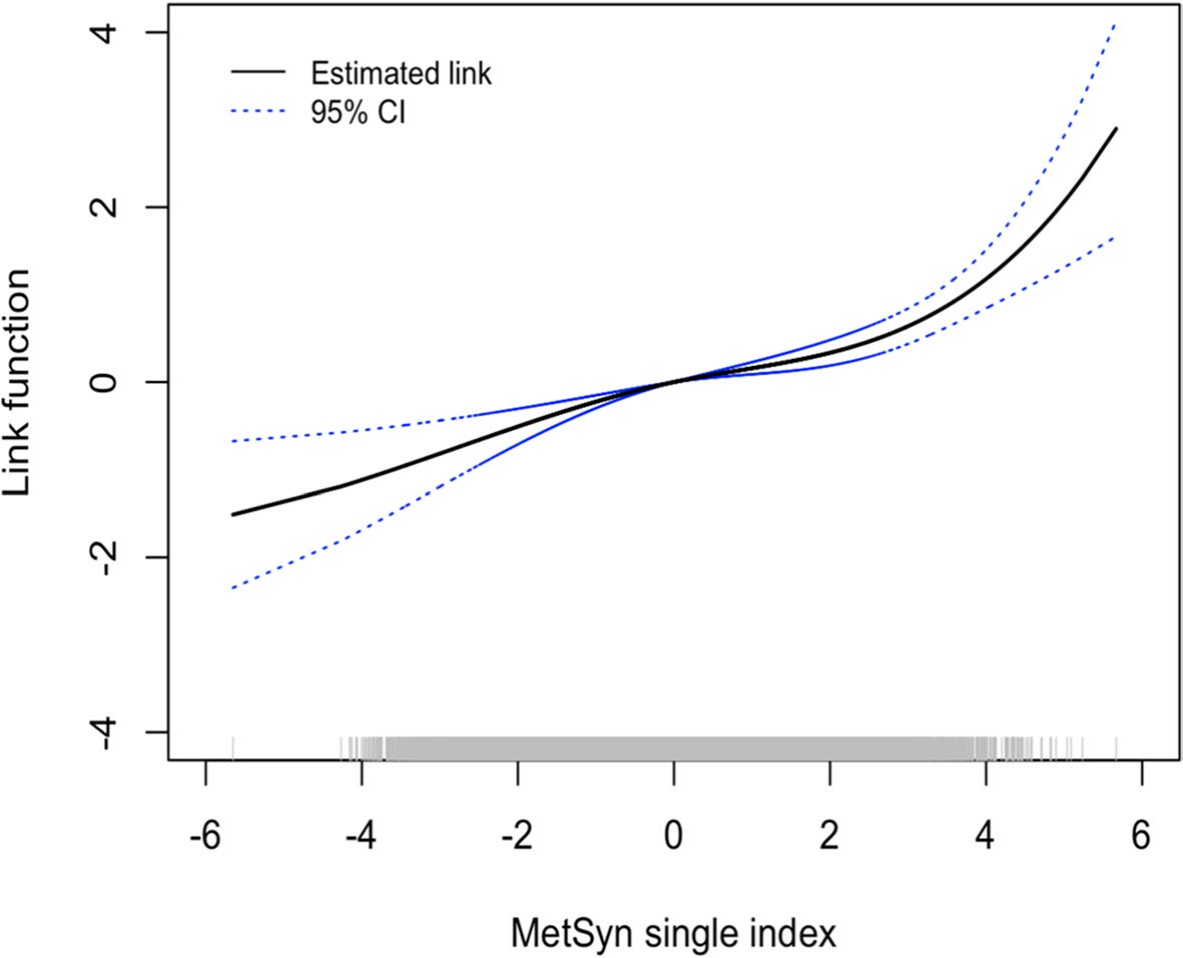
The estimated link function (solid, black) and 95% pointwise confidence interval (dotted, blue) for data application. The distribution of the single index value is indicated along the x-axis (grey) [Color figure online]. Adapted with permission of the American Thoracic Society. Copyright © 2023 American Thoracic Society. All rights reserved. Cite: Kwon S, Lee M, Crowley G, Schwartz T, Zeig-Owens R, Prezant DJ, Liu M, and Nolan A /2021 / Dynamic Metabolic Risk Profiling of World Trade Center Lung Disease: A Longitudinal Cohort Study /Am J Respir Crit Care Med / Vol 204(9) / 1035-1047. The American Journal of Respiratory and Critical Care Medicine is an official journal of the American Thoracic Society. Readers are encouraged to read the entire article for the correct context at [https://www.atsjournals.org/doi/full/10.1164/rccm.202006-2617OC]. The authors, editors, and The American Thoracic Society are not responsible for errors or omissions in adaptations

Figure 2 (adapted from Fig. 6A of Kwon et al. [16]) demonstrates a possible nonlinear joint effect of the MetSyn components on the survival risk. Because the estimated link function was monotone, we interpreted the joint effect qualitatively. When examining the extremes, having fewer MetSyn characteristics, and thereby being on the negative end of the spectrum of MetSyn single indices, had a modest effect on the risk of developing lung injury. On the other hand, when examining positive MetSyn single indices, the risk increased exponentially. For example, the HR of a 1-unit increase in the single index from 0 to 1 is 1.246 ($\:{=e}^{0.22-0.00})$, while the HR from 1 to 2 is 2.270 ($\:={e}^{1.04-0.22}$). When assessing the linearity of the single-index function using the LR test statistic, p-value=0.118 indicating that the overall relationship did not significantly deviate from linearity.

An advantage of using the proposed method is not only to provide the joint effects of multiple time-dependent covariates as a functional form $\:\psi\:(\bullet\:)$, but also to delineate the relative contribution weights with easy interpretability. Our data application led to an R shiny application promoting the utilization of metabolic syndrome in susceptible populations for dynamic risk assessment (https://med.nyu.edu/research/nolan-lab/software). In addition, the proposed method can handle multicollinearity and interactions, which are common issues when multiple covariates are under study [16, 22].

## Discussion

In cohort studies with survival outcomes, multiple time-dependent covariates are commonly observed; these can act synergistically or antagonistically on the risk of the event. However, classical methods such as time-dependent Cox regression model could encounter challenges with handling multiple time-dependent covariates because they (i) are inter-correlated, (ii) exhibit complex interactions, and (iii) involve possible non-linear relationships. Furthermore, quantifying the possible nonlinear joint effect of multiple covariates could improve our understanding of the disease mechanism in a real setting.

For studies involving multiple correlated covariates, several methods have been developed, such as weighted quantile sum (WQS) regression [23, 24] and Bayesian kernel machine regression (BKMR) [25, 26]. WQS regression is a parametric approach that assumes all exposures affect the outcome in the same direction, using a weighted sum score to estimate the overall linear effect. However, this assumption can be restrictive when the mechanisms of individual exposures are not fully understood [27]. BKMR is a nonparametric method that accommodates complex, nonlinear relationships between exposures and outcomes. However, its results can be hard to interpret and require large sample sizes, increasing computational demands [27]. Importantly, both WQS and BKMR have been adapted to investigate environmental exposures across diverse outcome types, such as WQS for longitudinal outcomes [28] and BKMR for time-to-event outcomes [29]. However, analytic methods remain limited in applying time-varying covariates within the Cox regression framework, which represents a methodological gap in modern cohort studies with survival outcomes.

The partial-linear single-index (PLSI) model is a natural extension of the partially linear model [30] and single-index model [31, 32] in which covariates can have both linear and nonlinear effects on the log hazard in the proportional hazards model. High-dimensional covariates with possible nonlinear effects can be first combined as a single index, providing a flexible and parsimonious model. This approach can reduce the dimensionality of the covariates through the single index and simultaneously provides efficient estimates of the covariate effects. While the features of multiple time-dependent covariates fit PLSI models well, previous studies of PLSI hazards models work only for time-independent covariates, making them less practical in modern studies. Note that PLSI techniques with time-dependent covariates have recently been applied to semiparametric transformation models with censored data by some of the authors [33].

Motivated by a real example from a longitudinal cohort study, we have developed a partial-linear single-index Cox regression model with multiple time-dependent covariates [16]. This model can be viewed as a natural extension of the traditional time-dependent Cox regression, enabling the investigation of both linear and nonlinear effects of the covariates. A B-spline smoothing technique and the maximum partial likelihood method are combined to feasibly obtain inferences about covariate effects and estimation of the nonparametric flexible function. We chose to use B-splines for their computational and theoretical advantages. We have shown that the proposed PLSI-Cox model performed better than the classical time-dependent Cox regression model when a nonlinear link function exists. Moreover, our novel method provides efficient estimation and clear interpretation. For example, coefficients of the covariates in the nonlinear components are interpreted as relative contribution weights, while estimates in the linear component have the usual interpretation as the log hazard ratio. Therefore, our proposed model is particularly effective for studying multiple, correlated covariates that may exhibit complex relationships, while also adjusting for confounding.

In our simulation study, we found that the proposed method exhibited robust performance under smaller sample sizes, highly correlated covariates, and censoring times generated by various mechanisms. (see Tables S1-S3 in Web Appendix C). Previous studies also demonstrated that the PLSI techniques performed robustly with various types of nonlinear relationships, including quadratic, sine curve, and log curve [13, 14, 20, 33–35]. Furthermore, using the LR statistic we examined type I error and power of our PLSI model, which have not been investigated previously in the literatures of the single-index models for survival outcomes [13, 14, 20]. It is worth noting that our bootstrapping approach to compute standard errors is practical for implementation, given the minimal computational burden of the PLSI-Cox model compared to the traditional Cox regression. For example, both methods performed within one second, even with a larger sample size (*N* = 5,000) considered (further details are provided in Web Appendix C).

In the analysis of the FDNY cohort study, we also conducted the Weibull proportional hazards (PH) model to investigate the impact of each exposure on lung injury [16]. Similar results were observed in both the Weibull PH model and the PLSI-Cox model; for example, BMI, triglycerides and HDL were significantly associated with the risk of lung injury. Compared to traditional PH modeling, the PLSI-Cox model not only enables us to rank the contributions of individual MetSyn characteristics to the risk of developing lung injury, but also offers an additional advantage by providing the nonlinear joint effects of all metabolic syndrome components, without concerns about multicollinearity and interactions between potentially highly correlated characteristics, such as SBP and DBP. This application facilitates the interpretation of the PLSI-Cox models and provides a platform for further assessing how individual components impact the MstSyn single-index and hazard ratios – for example, how reducing MetSyn factor lowers the likelihood of lung injury in susceptible populations. Furthermore, the weighted sum of risk from each component of MetSyn (i.e., single-index values) to calculate a cumulative risk score can be interpreted as a surrogate of severity of MetSyn using actual values and preserving information for future intervention (e.g., dietary) studies [16]. Thus, our methods can be generalized to study multiple longitudinal covariates across diseases that may exhibit nonlinear effects, which is a critical gap in modeling repeatedly measured exposures and evaluating their nonlinear joint effects on the risk of the event.

To establish the asymptotic properties, one can assume either fixed knots or an increasing number of knots [36]. In this manuscript, we assumed the first approach, and the bias caused by spline approximation is known to be relatively small compared to the variance of the estimated function [13, 14, 20, 36]. Given this assumption, we showed that our estimator behaves similarly (i.e., consistency and asymptotically normality) as the traditional Cox PH regression coefficients (Web Appendix B). Alternatively, the second approach does not assume the unknown function being a spline function. In this case, the number of knots must increase as the sample size increases. Furthermore, Wang [11] provided a large-sample theory under the proportional hazards regression models with unknown link function $\:g\left(\bullet\:\right)$, that is, $\:\lambda\:\left(t\right)={\lambda\:}_{0}\left(t\right)g\left({\beta\:}^{T}X\left(t\right)\right)$, where our proposed model can be seen as a special case. Such a development appeared to provide a reasonably good approximation in our simulation study. To select the number of knots, we suggest testing multiple knot configurations and choosing the best one based on a criterion such as AIC, BIC, or through a cross-validation procedure. We empirically confirmed that the results of our model estimations were not sensitive to the number of knots, aligning with previous studies [13, 14, 20, 33, 34].

This study has several limitations. First, the PLSI-Cox model requires the assumption of no interactions between $\:X\left(t\right)$ and $\:Z\left(t\right)$, which may be strong. However, in biomedical research—especially when exposures (e.g., environmental chemicals or biomarkers) are high-dimensional and highly correlated, but each individual exposure has a small impact—it is of interest to model their joint effects while adjusting for confounders to be linear effects [25, 26, 33–35, 37]. Such studies assume that $\:X$ includes all potential nonlinear exposures, while $\:Z$ includes confounding variables (e.g., patient demographics and socioeconomic status) that are pre-specified based on prior knowledge. Furthermore, since our methods incorporate a flexible functional form for exposures, they are robust to misspecifications in the relationships among exposures, offering an important direction for future research. When our interest lies on the interaction between two types of the covariates, varying index coefficients models, such as $\:{\sum\:}_{j=1}^{q}{\psi\:}_{j}\left({\beta\:}_{j}^{T}X\right){Z}_{j}$, can be considered [38, 39]. Such varying index coefficient model structures allow us to account for possible correlations between $\:X\left(t\right)$ and $\:Z\left(t\right)$. Since varying index coefficient models for survival outcomes are underexplored (to the best of our knowledge), we leave this topic as a direction for future research. Second, environmental health studies (as well as studies in other fields) often encounter complex exposures with natural groupings (i.e., multiple-index structures), such as phthalates, phenols, and metals. McGee et al. [40] recently proposed Bayesian multiple index models that account for non-linear and non-additive relationships between multiple exposure groupings and a continuous health outcome. This approach combines the strengths of response-surface methods, such as BKMR [25, 26], and exposure-index methods, such as WQS regression [23, 24] and single-index models [31, 36, 41–44]. Similarly, frequentist multiple-index modeling techniques could enhance our PLSI-Cox models, such as $\:\lambda\:\left(t\right)={\lambda\:}_{0}\left(t\right)\text{exp}\left\{{\sum\:}_{m=1}^{M}{\psi\:}_{m}\left({\beta\:}_{m}^{T}{X}_{m}\right)+{\alpha\:}^{T}Z\right\}$, where each $\:{X}_{m}$-vector covariate represents a mutually exclusive group based on scientific grouping information. These multiple-index modeling approaches for various types of health outcomes are currently being investigated by some of the authors. In such studies, we will further examine the robustness of the proposed estimator for the PLSI-Cox model when the true model includes interactions between $\:X$ and $\:Z$, multiple-index specifications, or both. Third, our proposed model assumes that the hazard depends only on the current covariates at time $\:t$, while the cumulative hazard and survival functions are conditional on the covariate history up to $\:t$. As a result, the hazard ratio in the PLSI-Cox regression model reflects concurrent effects. Further investigation into potential tests for lagged effects would be valuable for future implementation. Fourth, an additional assumption of our proposed method is that time-dependent covariates are external. In survival analysis, time-dependent covariates can be categorized in two different ways: external (or exogenous) and internal (or endogenous) [45]. As a classical method, time-dependent Cox regression models apply only for external covariates, while joint models have been developed to handle internal covariates [45, 46]. Because our proposed PLSI model assumed external covariates, we herein describe a further direction of the PLSI survival model under the joint modeling framework to incorporate multiple and internal time-dependent covariates. These future directions offer opportunities to further enhance our proposed PLSI survival models, enabling a more comprehensive analysis of potentially time-varying covariates in relation to survival outcomes in modern longitudinal studies.

## Supplementary Information

Supplementary Material 1.

## Data Availability

The R code is available from author’s GitHub (https://github.com/ml5977/plsi_survival_models). Data are available as per original publication. Restrictions apply to the availability of these data.
