# Supplementary material for: Partial-linear single-index Cox regression models with multiple time-dependent covariates
Source: BMC Med Res Methodol. 2024 Dec 20;24:311. doi: 10.1186/s12874-024-02434-9 (PMC11661057; doi:10.1186/s12874-024-02434-9)
Supplement: Supplementary file 1 — Supplementary Material 1. [file 12874_2024_2434_MOESM1_ESM.docx]

**Supporting Information for “Partial-linear single-index Cox regression models with multiple time-dependent covariates” by Lee et al.**

**Web Appendix A**. *Derivation of the marginal score functions and Hessian matrices of* $\left( \gamma,\beta, \alpha\right)$

Based on the equation (1) of the manuscript, the log-partial likelihood can be specified as

$$l\left( \theta\right)=\sum_{i=1}^{n} \Delta_{i}\left[ \gamma^{T}\tilde{B}\left( \beta^{T}X_{i}\left( T_{i} \right) \right)+\alpha^{T}Z_{i}\left( T_{i} \right)-\log\left( \sum_{j=1}^{n} Y_{j}\left( T_{i} \right)\exp\left( \gamma^{T}\tilde{B}\left( \beta^{T}X_{j}\left( T_{i} \right) \right)+\alpha^{T}Z_{j}(T_{i}) \right) \right) \right],$$

where $Y_{j}\left( t \right)=I\left( T_{j}\geq t \right)$. The score functions are

$S_{\gamma}=\frac{dl}{d\gamma}=\sum_{i=1}^{n} \Delta_{i}\left[ \tilde{B}\left( \beta^{T}X_{i}\left( T_{i} \right) \right)-\sum_{l=1}^{n} w_{li}\tilde{B}\left( \beta^{T}X_{l}\left( T_{i} \right) \right) \right],$ $S_{\beta}=\frac{dl}{d\beta}=\sum_{i=1}^{n} \Delta_{i}\left[ \gamma^{T}B\left( \beta^{T}X_{i}\left( T_{i} \right) \right)X_{i}^{T}\left( T_{i} \right)-\sum_{l=1}^{n} w_{li}\cdot\gamma^{T}B\left( \beta^{T}X_{l}\left( T_{i} \right) \right)X_{l}^{T}\left( T_{i} \right) \right],$ and $S_{\alpha}=\frac{dl}{d\alpha}=\sum_{i=1}^{n} \Delta_{i}\left[ Z_{i}\left( T_{i} \right)-\sum_{l=1}^{n} w_{li}Z_{l}\left( T_{i} \right) \right],$ where $w_{li}=\frac{Y_{l}\left( T_{i} \right)\exp\left( \gamma^{T}\tilde{B}\left( \beta^{T}X_{l}\left( T_{i} \right) \right)+\alpha^{T}Z_{l}\left( T_{i} \right) \right)}{\sum_{j=1}^{n} Y_{j}\left( T_{i} \right)\exp\left( \gamma^{T}\tilde{B}\left( \beta^{T}X_{j}\left( T_{i} \right) \right)+\alpha^{T}Z_{j}\left( T_{i} \right) \right)}$. The components of the Hessian matrix are

$H_{\beta,\beta}=\frac{d^{2}l}{d\beta d\beta^{T}}=\sum_{i=1}^{n} \Delta_{i}\left[ \gamma^{T}B\left( \beta^{T}X_{i}\left( T_{i} \right) \right)X_{i}^{T}\left( T_{i} \right)X_{i}\left( T_{i} \right)-\sum_{l=1}^{n} w_{li}\left( \gamma^{T}B\left( \beta^{T}X_{l}\left( T_{i} \right) \right)+\left\{ \gamma^{T}B\left( \beta^{T}X_{l}\left( T_{i} \right) \right) \right\}^{2} \right)X_{l}^{T}\left( T_{i} \right)X_{l}\left( T_{i} \right)+\sum_{l=1}^{n} w_{li}\gamma^{T}B\left( \beta^{T}X_{l}\left( T_{i} \right) \right)X_{l}^{T}(T_{i})\sum_{l=1}^{n} w_{li}\gamma^{T}B\left( \beta^{T}X_{l}\left( T_{i} \right) \right)X_{l}^{T}(T_{i}) \right],$

$H_{\gamma,\gamma}=\frac{d^{2}l}{d\gamma d\gamma^{T}}=-\sum_{i=1}^{n} \Delta_{i}\left[ \sum_{l=1}^{n} w_{li}\tilde{B}\left( \beta^{T}X_{l}\left( T_{i} \right) \right)\tilde{B}^{T}\left( \beta^{T}X_{l}\left( T_{i} \right) \right)-\sum_{l=1}^{n} w_{li}Z_{l}\left( T_{i} \right)\sum_{l=1}^{n} w_{li}Z_{l}^{T}\left( T_{i} \right) \right],$

$H_{\alpha,\alpha}=\frac{d^{2}l}{d\alpha d\alpha^{T}}=-\sum_{i=1}^{n} \Delta_{i}\left[ \sum_{l=1}^{n} w_{li}Z_{l}\left( T_{i} \right)Z_{l}^{T}\left( T_{i} \right)-\sum_{l=1}^{n} w_{li}Z_{l}\left( T_{i} \right)\sum_{l=1}^{n} w_{li}Z_{l}^{T}\left( T_{i} \right) \right],$

$H_{\gamma,\alpha}=\frac{d^{2}l}{d\gamma d\alpha}=-\sum_{i=1}^{n} \Delta_{i}\left[ \sum_{l=1}^{n} w_{li}\tilde{B}\left( \beta^{T}X_{l}\left( T_{i} \right) \right)Z_{l}^{T}\left( T_{i} \right)-\sum_{l=1}^{n} w_{li}\tilde{B}\left( \beta^{T}X_{l}\left( T_{i} \right) \right)\sum_{l=1}^{n} w_{li}Z_{l}^{T}\left( T_{i} \right) \right],$

$H_{\beta,\alpha}=\frac{d^{2}l}{d\beta d\alpha}=-\sum_{i=1}^{n} \Delta_{i}\left[ \sum_{l=1}^{n} w_{li}\gamma^{T}B\left( \beta^{T}X_{l}\left( T_{i} \right) \right)Z_{l}\left( T_{i} \right)X_{l}^{T}\left( T_{i} \right)-\sum_{l=1}^{n} w_{li}Z_{l}^{T}\left( T_{i} \right)\sum_{l=1}^{n} w_{li}\gamma^{T}B\left( \beta^{T}X_{l}\left( T_{i} \right) \right)X_{l}^{T}\left( T_{i} \right) \right],$ and

$H_{\beta,\gamma}=\frac{d^{2}l}{d\beta d\gamma}=\sum_{i=1}^{n} \Delta_{i}\left[ B\left( \beta^{T}X_{i}\left( T_{i} \right) \right)X_{i}^{T}\left( T_{i} \right)-\sum_{l=1}^{n} w_{li}\left( 1+\gamma^{T}\tilde{B}\left( \beta^{T}X_{l}\left( T_{i} \right) \right) \right)B\left( \beta^{T}X_{l}\left( T_{i} \right) \right)X_{l}^{T}\left( T_{i} \right)+\sum_{l=1}^{n} w_{li}\tilde{B}\left( \beta^{T}X_{l}\left( T_{i} \right) \right)\sum_{l=1}^{n} w_{li}\gamma^{T}B\left( \beta^{T}X_{l}\left( T_{i} \right) \right)X_{l}^{T}\left( T_{i} \right) \right].$

**Web Appendix B**. *Asymptotic results using the sandwich formula*

Applying standard counting process theory [1, 2], we studied the asymptotic properties of the estimate of the regression coefficients and the spline coefficients. We assumed pre-specified and fixed number of knots for simplicity. Liu [3] studied the large sample properties when the covariates are time-independent under the single index model (i.e., $\lambda\left( t \right)=\lambda_{0}\left( t \right)\exp\left\{ \psi\left( \beta^{T}X \right) \right\}$); our work is the extension of Huang and Liu [4] and Liu [3] for the PLSI model. Thus, we used same notations, processes of $S^{\left( l \right)}, l=0,\ldots,7$, and conditions (1) to (6) from Liu [3], but with a combined covariate process, $g\left( W\left( t \right);\theta\right)=\sum_{k=1}^{K} \gamma_{k}\tilde{B}_{k}\left( {X\left( t \right)}^{T}\beta\left( \sigma\right) \right)+{Z\left( t \right)}^{T}\alpha$ with $\theta=\left( \gamma, \sigma, \alpha\right)$ where $W\left( t \right)$ includes both $X\left( t \right)$ and $Z\left( t \right)$; $\beta\left( \sigma\right)=\left( \left( 1-\left\| \sigma\right\|^{2} \right)^{1/2}, \sigma_{1},\ldots,\sigma_{p-1} \right)^{T}$with $\sigma=\left( \sigma_{1},\ldots,\sigma_{p-1} \right)^{T}$; and $\tilde{B}_{k},k=1,\ldots,K,$ are the integrals of the B-spline basis functions as defined. In Web Appendix B, for a column vector $a$, we denote $a^{\otimes2}$ for the matrix $aa^{T}$, $\left\| a \right\|=\sup_{i} a_{i}$, and $\left| a \right|=\left( a^{T}a \right)^{1/2}$. For a matrix $A$, we denote $\left\| A \right\|=\sup_{i,j} a_{ij}$. For a function $g\left( w;\theta\right)$, let $\dot{g}\left( w;\theta\right)$ and $\ddot{g}\left( w;\theta\right)$ denote the gradient and Hessian of $g$ relative to $\theta$. Borrowing from Liu [3], we define

$$S^{\left( 0 \right)}\left( \theta, t \right)=\frac{1}{n}\sum_{i=1}^{n} Y_{i}\left( t \right)\exp\left\{ g\left( W_{i}\left( t \right);\theta\right) \right\},$$

$$S^{\left( 1 \right)}\left( \theta, t \right)=\frac{1}{n}\sum_{i=1}^{n} \dot{g}\left( W_{i}\left( t \right);\theta\right)Y_{i}\left( t \right)\exp\left\{ g\left( W_{i}\left( t \right);\theta\right) \right\},$$

$$S^{\left( 2 \right)}\left( \theta, t \right)=\frac{1}{n}\sum_{i=1}^{n} \dot{g}\left( W_{i}\left( t \right);\theta\right)\dot{g^{T}}\left( W_{i}\left( t \right);\theta\right)Y_{i}\left( t \right)\exp\left\{ g\left( W_{i}\left( t \right);\theta\right) \right\},$$

$$S^{\left( 3 \right)}\left( \theta, t \right)=\frac{1}{n}\sum_{i=1}^{n} \left\{ \ddot{g}\left( W_{i}\left( t \right);\theta\right)+\dot{g}\left( W_{i}\left( t \right);\theta\right)\dot{g^{T}}\left( W_{i}\left( t \right);\theta\right) \right\}Y_{i}\left( t \right)\exp\left\{ g\left( W_{i}\left( t \right);\theta\right) \right\},$$

$$S^{\left( 4 \right)}\left( \theta, t \right)=\frac{1}{n}\sum_{i=1}^{n} \left\{ g\left( W_{i}\left( t \right);\theta\right)-g\left( W_{i}\left( t \right);\theta_{0} \right) \right\}Y_{i}\left( t \right)\exp\left\{ g\left( W_{i}\left( t \right);\theta_{0} \right) \right\},$$

$$S^{\left( 5 \right)}\left( \theta, t \right)=\frac{1}{n}\sum_{i=1}^{n} \dot{g}\left( W_{i}\left( t \right);\theta\right)Y_{i}\left( t \right)\exp\left\{ g\left( W_{i}\left( t \right);\theta_{0} \right) \right\},$$

$$S^{\left( 6 \right)}\left( \theta, t \right)=\frac{1}{n}\sum_{i=1}^{n} \ddot{g}\left( W_{i}\left( t \right);\theta\right)Y_{i}\left( t \right)\exp\left\{ g\left( W_{i}\left( t \right);\theta_{0} \right) \right\},$$

$$S^{\left( 7 \right)}\left( \theta, t \right)=\frac{1}{n}\sum_{i=1}^{n} \dot{g}\left( W_{i}\left( t \right);\theta\right)\dot{g^{T}}\left( W_{i}\left( t \right);\theta\right)Y_{i}\left( t \right)\exp\left\{ g\left( W_{i}\left( t \right);\theta_{0} \right) \right\},$$

$E\left( \theta, t \right)=S^{\left( 1 \right)}\left( \theta, t \right)/S^{\left( 0 \right)}\left( \theta, t \right)$ and $V\left( \theta, t \right)=S^{\left( 2 \right)}\left( \theta, t \right)/S^{\left( 0 \right)}\left( \theta, t \right)-E\left( \theta, t \right)^{\otimes2}.$

The six conditions are follows by Liu [3]:

1. (Finite interval). $\int_{0}^{1} \lambda_{0}\left( t \right)dt<\infty$
2. (Asymptotic stability). There exists a compact neighborhood $\Theta$ of $\theta_{0}$ and functions $s^{(i)}, i=0, 1,\ldots, 7$, defined on $\Theta\times\left[ 0,1 \right]$ such that for $i=0, 1,\ldots, 7$,

$$\sup_{t\in\left[ 0,1 \right], \theta\in\Theta} \left\| S^{\left( i \right)}\left( \theta, t \right)-s^{\left( i \right)}\left( \theta, t \right) \right\|\to_{p}0.$$

3. (Lindeberg condition). For any $\epsilon>0$ and $j=1,2,\ldots,\left( p+q \right)$,

$$\int_{0}^{1} n^{-1}\sum_{i=1}^{n} \left[ \dot{g}\left( W_{ij}\left( t \right), \theta_{0} \right)-E\left( \theta_{0}, t \right)_{j} \right]^{2}Y_{i}\left( t \right)\exp\left\{ g\left( W_{i}\left( t \right);\theta_{0} \right) \right\}I_{\left\{ n^{-1/2}|\dot{g}\left( W_{ij}\left( t \right), \theta_{0} \right)-E\left( \theta_{0}, t \right)_{j}>\epsilon\right\}}\lambda_{0}\left( t \right)dt\to_{p}0$$

4. (Asymptotic regularity conditions). Let $e=s^{\left( 1 \right)}/s^{(0)}$ and $v=s^{(2)}/s^{(0)}-e^{\otimes2}$. For each $j=0,1,\ldots,7,$ $s^{\left( j \right)}\left( \cdot, t \right)$ are continuous functions of $\theta\in\Theta$, uniformly in $t\in\left[ 0,1 \right]$. Also, $s^{\left( j \right)}, j=0,1,\ldots,7$, are bounded on $\Theta\times\left[ 0,1 \right], s^{\left( 0 \right)}$ is bounded away from zero and the matrix $\Sigma=\int_{0}^{1} v\left( \theta_{0}, t \right)s^{\left( 0 \right)}\left( \theta_{0}t \right)\lambda_{0}\left( t \right)dt$ is positive definite. In addition, $s^{\left( 0 \right)}\left( \theta, t \right)$ and $s^{\left( 4 \right)}\left( \theta, t \right)$ are assumed to be twice differentiable with respect to $\theta$ on $\Theta\times\left[ 0,1 \right]$.
5. $\dot{g}\left( W_{i}\left( t \right);\theta\right)$ is locally bounded for $i=1,\ldots,n$, $j=1,\ldots,\left( p+q \right)$.

We recommend that readers carefully examine the further details of the large sample property in Liu [3]. Let $N$ be a counting process with continuous compensator $A$ and let $M=N-A$ be a martingale process. We directly provide two theorems with their brief proofs as follows.

**Theorem 1** (Consistency of $\hat{\theta}).$ There exists a sequence of roots $\hat{\theta}_{n}$ of the partial likelihood equation such that $\hat{\theta}_{n}\to_{p}\theta_{0}$.

*Proof*. Let $Z_{n}\left( \theta,t \right)=\frac{1}{n}\left[ \log L\left( \theta,t \right)-\log L\left( \theta_{0},t \right) \right]$ and

$$A_{n}\left( \theta,t \right)=\frac{1}{n}\left[ \sum_{i=1}^{n} \int_{0}^{t} \left\{ g\left( W_{i}\left( s \right);\theta\right)-g\left( W_{i}\left( s \right);\theta_{0} \right) \right\}\lambda_{i}\left( s \right)ds-\int_{0}^{t} \log\frac{S^{\left( 0 \right)}\left( \theta,s \right)}{S^{\left( 0 \right)}\left( \theta_{0},s \right)}\bar{\lambda}(s)ds \right]$$

where $\bar{\lambda}=\sum_{i=1}^{n} \lambda_{i}$. Then the process

$$Z_{n}\left( \theta,t \right)-A_{n}\left( \theta,t \right)=\frac{1}{n}\left[ \sum_{i=1}^{n} \int_{0}^{t} \left\{ g\left( W_{i}\left( s \right);\theta\right)-g\left( W_{i}\left( s \right);\theta_{0} \right)-\log\frac{S^{\left( 0 \right)}\left( \theta,s \right)}{S^{\left( 0 \right)}\left( \theta_{0},s \right)} \right\}d\bar{M}_{i}\left( s \right) \right]$$

is a locally square integrable martingale for $\theta$, with predictable variation process at $t$ given by $\left\langle Z_{n}\left( \theta,\cdot\right)-A_{n}\left( \theta,\cdot\right), Z_{n}\left( \theta,\cdot\right)-A_{n}\left( \theta,\cdot\right) \right\rangle\left( t \right)$, which converges to zero in probability (proof is similar to Liu [3]). By conditions (1), (2), and (4), the Taylor expansion, and the Cauchy-Schwarz inequality, $\left\langle Z_{n}\left( \theta,\cdot\right)-A_{n}\left( \theta,\cdot\right), Z_{n}\left( \theta,\cdot\right)-A_{n}\left( \theta,\cdot\right) \right\rangle\left( t \right)$ converges to zero in probability. Thus, by Lemma 1[3], $\lim_{n\to\infty} \left\{ Z_{n}\left( \theta,t \right)-A_{n}(\theta,t) \right\}=0$ in probability for all $\theta\in\Theta$. Because $\Theta$ is a compact set, $Z_{n}(\theta,t)$ converges to $A_{n}(\theta,t)$ in probability uniformly for $\theta\in\Theta$. On the other hand, from conditions (1), (2), and (4), $\theta_{0}$ is a local maximizer of $A(\theta,1)$. Because $\lim_{n\to\infty} \left\{ Z_{n}\left( \theta,1 \right)-A\left( \theta,1 \right) \right\}=0$ in probability uniformly for $\theta\in\Theta_{1}$, the maximizer $\hat{\theta}$ of $Z_{n}(\theta,1)$ on $\Theta_{1}$ will converge to $\theta_{0}$. Because $\hat{\theta}$ should lie in the interior of $\Theta_{1}$ for large $n$, it solves the partial likelihood equation (i.e., $\hat{\theta}_{n}$). Thus, we have shown the existence of a sequence of consistent roots of the partial likelihood equation.

**Theorem 2** (Asymptotic normality of $\hat{\theta}$). There exists a sequence of roots $\hat{\theta}_{n}$ of the partial likelihood equation such that $n^{1/2}\left( \hat{\theta}_{n}-\theta_{0} \right)\to_{D}N(0, \Sigma^{-1}V\Sigma^{-1}).$

*Proof*. By the Taylor expansion for the score function of $\theta$ (i.e., $U\left( \theta, t \right)$), $U\left( \theta,1 \right)-U\left( \theta_{0},1 \right)=-H\left( \theta^{*},1 \right)\left( \theta-\theta_{0} \right),$ where $\theta^{*}$ lies between $\theta$ and $\theta_{0}$ and $H\left( \theta,t \right)=\int_{0}^{t} \sum_{i=1}^{n} \left\{ -g^{''}\left( W_{i}\left( s \right);\theta\right)+\frac{S^{\left( 3 \right)}\left( \theta,s \right)}{S^{\left( 0 \right)}\left( \theta,s \right)}-\left( \frac{S^{\left( 1 \right)}\left( \theta,s \right)}{S^{\left( 0 \right)}(\theta,s)} \right)^{\bigotimes2} \right\}dN_{i}\left( s \right).$ Hence, at $\theta=\hat{\theta}$, $n^{-1}H\left( \theta^{*},1 \right)\sqrt{n}\left( \hat{\theta}-\theta_{0} \right)=n^{-1/2}U\left( \theta_{0},1 \right).$ If we think of $\hat{\theta}$ as an estimator of $\theta$, then the covariance matrix of $\hat{\theta}$ can be estimated by the sandwich formula [5-8]. Define $\Sigma=\sum_{i=1}^{n} \frac{\partial}{\partial\theta}l_{i}\left( \theta\right)$ and $V=\sum_{i=1}^{n} l_{i}\left( \theta\right)l_{i}\left( \theta\right)^{T}$. Because $n^{-1/2}U\left( \theta_{0},1 \right)\to_{D}N(0, V)$ and $n^{-1}H\left( \theta^{*},1 \right)\to_{p}\Sigma$ (proof is similar to Liu [3]), $n^{\frac{1}{2}}\left( \hat{\theta}_{n}-\theta_{0} \right)\to_{D}N(0, \Sigma^{-1}V\Sigma^{-1})$ by the Slutsky theorem.

**Web Appendix C**. *Additional simulation results*

In our simulation study, we conducted additional simulations to evaluate the robustness of our proposed models under various scenarios: a small sample size with N=200 (Table S1), different censoring mechanisms (Table S2), and high correlations ($\rho=0.9$) between exposures (Table S3). For the various censoring mechanisms, we generated censoring times from exponential and uniform distributions, using parameters chosen to achieve 25% and 50% censoring rates. To compute standard errors and the coverage probability of the 95% confidence intervals, we used 200 bootstrap samples. Simulation results are provided in the below tables.

From the perspective of computational cost, the proposed iterative estimation procedure involves fitting time-dependent Cox regression multiple times during iterations. Therefore, it is not surprising that our proposed methods entail a higher computational cost compared to traditional time-dependent Cox regression. However, this cost was found to be minimal. For instance, the average computation time for the PLSI-Cox model was 0.10 seconds (SD = 0.06), compared to 0.01 seconds (SD = 0.001) for the traditional time-dependent Cox regression with a sample size of 500. When the sample size was increased to 5,000, the PLSI-Cox model and the traditional time-dependent Cox regression required 0.54 seconds (SD = 0.08) and 0.09 seconds (SD = 0.01), respectively. The increase in computational cost with sample size was comparable for both methods, and both completed the analyses within one second.

**Table S1**. Simulation results with small sample sizes (N=200)

|  | Time-dependent Cox model | | | | Proposed PLSI-Cox model | | | |
| --- | --- | --- | --- | --- | --- | --- | --- | --- |
|  | Bias | SD | SE | CP | Bias | SD | SE | CP |
|  | Linear model with censoring rate 25% (100% converged) | | | | | | | |
| $\beta_{1}$ | -0.004 | 0.056 | 0.060 | 0.940 | -0.004 | 0.059 | 0.067 | 0.950 |
| $\beta_{2}$ | 0.008 | 0.051 | 0.060 | 0.960 | 0.006 | 0.054 | 0.068 | 0.980 |
| $\beta_{3}$ | -0.005 | 0.058 | 0.060 | 0.945 | -0.005 | 0.060 | 0.067 | 0.960 |
| $\beta_{4}$ | 0.007 | 0.058 | 0.060 | 0.960 | 0.010 | 0.059 | 0.068 | 0.975 |
| $\beta_{5}$ | -0.004 | 0.064 | 0.059 | 0.915 | -0.003 | 0.064 | 0.069 | 0.940 |
| $\beta_{6}$ | -0.001 | 0.065 | 0.060 | 0.915 | 0.001 | 0.066 | 0.068 | 0.930 |
| $\beta_{7}$ | 0.004 | 0.059 | 0.060 | 0.960 | 0.004 | 0.060 | 0.069 | 0.950 |
| $\beta_{8}$ | -0.008 | 0.059 | 0.060 | 0.945 | -0.010 | 0.060 | 0.068 | 0.970 |
| $\alpha_{1}$ | 0.22 | 0.089 | 0.101 | 0.965 | 0.028 | 0.090 | 0.106 | 0.980 |
| $\alpha_{2}$ | -0.020 | 0.093 | 0.100 | 0.950 | -0.028 | 0.094 | 0.105 | 0.955 |
| $\alpha_{3}$ | 0.022 | 0.179 | 0.198 | 0.970 | 0.027 | 0.181 | 0.208 | 0.970 |
|  | Linear model with censoring rate 50% (100% converged) | | | | | | | |
| $\beta_{1}$ | -0.003 | 0.073 | 0.072 | 0.945 | -0.006 | 0.076 | 0.084 | 0.980 |
| $\beta_{2}$ | 0.009 | 0.073 | 0.072 | 0.945 | 0.009 | 0.078 | 0.089 | 0.975 |
| $\beta_{3}$ | 0.000 | 0.074 | 0.071 | 0.930 | -0.001 | 0.076 | 0.087 | 0.975 |
| $\beta_{4}$ | 0.006 | 0.07 | 0.072 | 0.935 | 0.005 | 0.081 | 0.088 | 0.970 |
| $\beta_{5}$ | -0.006 | 0.078 | 0.071 | 0.920 | -0.007 | 0.081 | 0.086 | 0.945 |
| $\beta_{6}$ | -0.012 | 0.071 | 0.072 | 0.955 | -0.015 | 0.075 | 0.088 | 0.970 |
| $\beta_{7}$ | 0.012 | 0.073 | 0.072 | 0.910 | 0.015 | 0.079 | 0.088 | 0.950 |
| $\beta_{8}$ | -0.013 | 0.067 | 0.072 | 0.955 | -0.012 | 0.071 | 0.087 | 0.965 |
| $\alpha_{1}$ | 0.076 | 0.116 | 0.131 | 0.955 | 0.087 | 0.120 | 0.143 | 0.955 |
| $\alpha_{2}$ | -0.060 | 0.121 | 0.130 | 0.950 | -0.070 | 0.125 | 0.141 | 0.955 |
| $\alpha_{3}$ | 0.039 | 0.215 | 0.258 | 0.990 | 0.043 | 0.225 | 0.285 | 0.990 |
|  | Nonlinear model with censoring rate 25% (100% converged) | | | | | | | |
| $\beta_{1}$ | -0.346 | 0.358 | 0.255 | 0.650 | 0.001 | 0.031 | 0.034 | 0.970 |
| $\beta_{2}$ | 0.313 | 0.321 | 0.248 | 0.705 | 0.001 | 0.032 | 0.033 | 0.945 |
| $\beta_{3}$ | -0.342 | 0.335 | 0.249 | 0.640 | -0.005 | 0.031 | 0.035 | 0.980 |
| $\beta_{4}$ | 0.367 | 0.348 | 0.247 | 0.615 | 0.001 | 0.029 | 0.033 | 0.965 |
| $\beta_{5}$ | -0.334 | 0.336 | 0.246 | 0.680 | -0.002 | 0.030 | 0.034 | 0.960 |
| $\beta_{6}$ | -0.350 | 0.365 | 0.282 | 0.695 | -0.003 | 0.038 | 0.042 | 0.975 |
| $\beta_{7}$ | 0.353 | 0.364 | 0.276 | 0.695 | 0.001 | 0.034 | 0.037 | 0.955 |
| $\beta_{8}$ | -0.311 | 0.397 | 0.283 | 0.695 | 0.000 | 0.038 | 0.042 | 0.970 |
| $\alpha_{1}$ | -0.303 | 0.686 | 0.805 | 0.970 | 0.022 | 0.661 | 0.813 | 0.980 |
| $\alpha_{2}$ | 0.399 | 0.740 | 0.802 | 0.925 | -0.009 | 0.763 | 0.809 | 0.950 |
| $\alpha_{3}$ | -0.187 | 0.177 | 0.184 | 0.840 | 0.027 | 0.174 | 0.189 | 0.975 |
|  | Nonlinear model with censoring rate 50% (99.0% converged) | | | | | | | |
| $\beta_{1}$ | -0.366 | 0.330 | 0.245 | 0.625 | -0.004 | 0.049 | 0.057 | 0.970 |
| $\beta_{2}$ | 0.359 | 0.336 | 0.242 | 0.650 | 0.002 | 0.051 | 0.055 | 0.960 |
| $\beta_{3}$ | -0.388 | 0.332 | 0.245 | 0.585 | -0.003 | 0.049 | 0.057 | 0.965 |
| $\beta_{4}$ | 0.368 | 0.348 | 0.243 | 0.660 | 0.006 | 0.052 | 0.054 | 0.970 |
| $\beta_{5}$ | -0.318 | 0.357 | 0.244 | 0.625 | -0.006 | 0.047 | 0.056 | 0.960 |
| $\beta_{6}$ | -0.364 | 0.382 | 0.273 | 0.645 | -0.005 | 0.061 | 0.068 | 0.965 |
| $\beta_{7}$ | 0.367 | 0.378 | 0.270 | 0.675 | 0.000 | 0.053 | 0.063 | 0.970 |
| $\beta_{8}$ | -0.318 | 0.364 | 0.276 | 0.655 | -0.006 | 0.062 | 0.068 | 0.955 |
| $\alpha_{1}$ | -0.232 | 0.879 | 0.991 | 0.955 | 0.109 | 0.842 | 1.044 | 0.985 |
| $\alpha_{2}$ | 0.101 | 0.943 | 0.989 | 0.945 | -0.220 | 0.966 | 1.035 | 0.975 |
| $\alpha_{3}$ | -0.087 | 0.225 | 0.229 | 0.925 | 0.085 | 0.229 | 0.245 | 0.949 |

**Table S2**. Simulation results of PLSI-Cox model with different censoring mechanisms

| True | Linear model | | | | Nonlinear model | | | |
| --- | --- | --- | --- | --- | --- | --- | --- | --- |
|  | Bias | SD | SE | CP | Bias | SD | SE | CP |
|  | Censoring time ~ $exp$ with censoring rate 25% (99% converged) | | | | | | | |
| $\beta_{1}$ | -0.005 | 0.035 | 0.039 | 0.970 | -0.002 | 0.017 | 0.039 | 0.970 |
| $\beta_{2}$ | -0.001 | 0.037 | 0.039 | 0.955 | -0.001 | 0.013 | 0.039 | 0.940 |
| $\beta_{3}$ | 0.002 | 0.039 | 0.039 | 0.930 | -0.002 | 0.017 | 0.039 | 0.925 |
| $\beta_{4}$ | 0.005 | 0.039 | 0.039 | 0.940 | 0.001 | 0.016 | 0.039 | 0.920 |
| $\beta_{5}$ | -0.003 | 0.040 | 0.039 | 0.945 | 0.000 | 0.016 | 0.039 | 0.930 |
| $\beta_{6}$ | 0.002 | 0.037 | 0.039 | 0.940 | 0.000 | 0.019 | 0.039 | 0.970 |
| $\beta_{7}$ | 0.006 | 0.039 | 0.039 | 0.960 | 0.000 | 0.018 | 0.039 | 0.950 |
| $\beta_{8}$ | -0.001 | 0.035 | 0.039 | 0.985 | 0.002 | 0.018 | 0.039 | 0.980 |
| $\alpha_{1}$ | -0.022 | 0.051 | 0.057 | 0.945 | 0.025 | 0.448 | 0.057 | 0.960 |
| $\alpha_{2}$ | 0.021 | 0.047 | 0.057 | 0.965 | 0.061 | 0.494 | 0.057 | 0.965 |
| $\alpha_{3}$ | -0.022 | 0.100 | 0.115 | 0.975 | -0.020 | 0.113 | 0.115 | 0.965 |
|  | Censoring time ~ $exp$ with censoring rate 50% (99% converged) | | | | | | | |
| $\beta_{1}$ | -0.008 | 0.048 | 0.046 | 0.935 | 0.001 | 0.022 | 0.046 | 0.935 |
| $\beta_{2}$ | 0.005 | 0.046 | 0.046 | 0.940 | 0.001 | 0.024 | 0.046 | 0.935 |
| $\beta_{3}$ | 0.004 | 0.041 | 0.046 | 0.975 | -0.004 | 0.025 | 0.046 | 0.985 |
| $\beta_{4}$ | 0.002 | 0.045 | 0.046 | 0.950 | 0.001 | 0.023 | 0.046 | 0.940 |
| $\beta_{5}$ | -0.003 | 0.045 | 0.046 | 0.940 | -0.004 | 0.022 | 0.045 | 0.925 |
| $\beta_{6}$ | -0.002 | 0.045 | 0.046 | 0.955 | 0.002 | 0.026 | 0.046 | 0.935 |
| $\beta_{7}$ | 0.007 | 0.047 | 0.046 | 0.925 | -0.001 | 0.025 | 0.046 | 0.925 |
| $\beta_{8}$ | 0.000 | 0.046 | 0.046 | 0.955 | 0.000 | 0.027 | 0.046 | 0.955 |
| $\alpha_{1}$ | 0.023 | 0.073 | 0.072 | 0.955 | 0.054 | 0.623 | 0.072 | 0.844 |
| $\alpha_{2}$ | -0.028 | 0.071 | 0.072 | 0.950 | -0.047 | 0.588 | 0.072 | 0.854 |
| $\alpha_{3}$ | 0.022 | 0.144 | 0.146 | 0.955 | 0.016 | 0.140 | 0.146 | 0.905 |
|  | Censoring time ~ $unif$ with censoring rate 25% (99% converged) | | | | | | | |
| $\beta_{1}$ | -0.001 | 0.042 | 0.038 | 0.915 | -0.002 | 0.016 | 0.038 | 0.925 |
| $\beta_{2}$ | 0.000 | 0.035 | 0.037 | 0.960 | 0.001 | 0.017 | 0.037 | 0.955 |
| $\beta_{3}$ | 0.000 | 0.040 | 0.038 | 0.925 | -0.001 | 0.017 | 0.038 | 0.930 |
| $\beta_{4}$ | 0.006 | 0.038 | 0.038 | 0.945 | 0.002 | 0.016 | 0.038 | 0.950 |
| $\beta_{5}$ | -0.003 | 0.038 | 0.037 | 0.945 | 0.000 | 0.017 | 0.038 | 0.940 |
| $\beta_{6}$ | -0.004 | 0.039 | 0.038 | 0.920 | 0.000 | 0.018 | 0.038 | 0.915 |
| $\beta_{7}$ | -0.001 | 0.040 | 0.038 | 0.940 | -0.001 | 0.019 | 0.038 | 0.945 |
| $\beta_{8}$ | -0.005 | 0.036 | 0.037 | 0.935 | 0.000 | 0.020 | 0.038 | 0.940 |
| $\alpha_{1}$ | -0.011 | 0.055 | 0.057 | 0.965 | -0.012 | 0.477 | 0.057 | 0.930 |
| $\alpha_{2}$ | 0.014 | 0.053 | 0.057 | 0.945 | 0.084 | 0.466 | 0.057 | 0.935 |
| $\alpha_{3}$ | -0.012 | 0.105 | 0.115 | 0.965 | -0.010 | 0.121 | 0.115 | 0.950 |
|  | Censoring time ~ $unif$ with censoring rate 50% (99% converged) | | | | | | | |
| $\beta_{1}$ | 0.003 | 0.043 | 0.045 | 0.935 | -0.002 | 0.027 | 0.045 | 0.940 |
| $\beta_{2}$ | 0.007 | 0.043 | 0.044 | 0.940 | 0.001 | 0.026 | 0.045 | 0.940 |
| $\beta_{3}$ | 0.000 | 0.041 | 0.045 | 0.960 | 0.000 | 0.028 | 0.045 | 0.965 |
| $\beta_{4}$ | 0.004 | 0.045 | 0.044 | 0.940 | 0.000 | 0.031 | 0.045 | 0.935 |
| $\beta_{5}$ | -0.006 | 0.047 | 0.045 | 0.945 | 0.000 | 0.025 | 0.045 | 0.940 |
| $\beta_{6}$ | 0.001 | 0.044 | 0.045 | 0.955 | -0.005 | 0.031 | 0.045 | 0.955 |
| $\beta_{7}$ | 0.006 | 0.047 | 0.045 | 0.935 | 0.002 | 0.030 | 0.045 | 0.920 |
| $\beta_{8}$ | -0.002 | 0.048 | 0.044 | 0.930 | 0.000 | 0.038 | 0.044 | 0.910 |
| $\alpha_{1}$ | 0.020 | 0.062 | 0.070 | 0.955 | -0.002 | 0.621 | 0.070 | 0.889 |
| $\alpha_{2}$ | -0.022 | 0.063 | 0.070 | 0.955 | -0.101 | 0.657 | 0.070 | 0.889 |
| $\alpha_{3}$ | 0.002 | 0.139 | 0.143 | 0.940 | 0.035 | 0.145 | 0.143 | 0.915 |

**Table S3**. Simulation results with high correlation settings (N=500)

|  | Time-dependent Cox model | | | | Proposed PLSI-Cox model | | | |
| --- | --- | --- | --- | --- | --- | --- | --- | --- |
|  | Bias | SD | SE | CP | Bias | SD | SE | CP |
|  | Linear model with censoring rate 25% (100% converged) | | | | | | | |
| $\beta_{1}$ | -0.020 | 0.102 | 0.101 | 0.930 | -0.020 | 0.103 | 0.102 | 0.930 |
| $\beta_{2}$ | 0.018 | 0.100 | 0.094 | 0.920 | 0.018 | 0.101 | 0.113 | 0.970 |
| $\beta_{3}$ | -0.015 | 0.105 | 0.100 | 0.940 | -0.014 | 0.107 | 0.125 | 0.960 |
| $\beta_{4}$ | 0.028 | 0.098 | 0.094 | 0.950 | 0.028 | 0.100 | 0.112 | 0.965 |
| $\beta_{5}$ | -0.011 | 0.102 | 0.100 | 0.935 | -0.012 | 0.105 | 0.122 | 0.950 |
| $\beta_{6}$ | -0.010 | 0.111 | 0.100 | 0.915 | -0.012 | 0.113 | 0.127 | 0.930 |
| $\beta_{7}$ | 0.011 | 0.088 | 0.094 | 0.950 | 0.013 | 0.091 | 0.114 | 0.980 |
| $\beta_{8}$ | -0.006 | 0.107 | 0.100 | 0.930 | -0.007 | 0.108 | 0.124 | 0.945 |
| $\alpha_{1}$ | 0.005 | 0.054 | 0.056 | 0.935 | 0.007 | 0.054 | 0.057 | 0.950 |
| $\alpha_{2}$ | -0.006 | 0.050 | 0.056 | 0.980 | -0.008 | 0.050 | 0.057 | 0.975 |
| $\alpha_{3}$ | 0.007 | 0.116 | 0.112 | 0.940 | 0.008 | 0.116 | 0.113 | 0.940 |
|  | Linear model with censoring rate 50% (100% converged) | | | | | | | |
| $\beta_{1}$ | -0.024 | 0.018 | 0.110 | 0.945 | -0.024 | 0.109 | 0.113 | 0.930 |
| $\beta_{2}$ | 0.011 | 0.114 | 0.105 | 0.935 | 0.011 | 0.119 | 0.132 | 0.955 |
| $\beta_{3}$ | -0.037 | 0.122 | 0.113 | 0.900 | -0.038 | 0.124 | 0.143 | 0.940 |
| $\beta_{4}$ | 0.013 | 0.110 | 0.106 | 0.940 | 0.012 | 0.116 | 0.130 | 0.945 |
| $\beta_{5}$ | 0.003 | 0.116 | 0.111 | 0.940 | 0.003 | 0.122 | 0.145 | 0.960 |
| $\beta_{6}$ | -0.042 | 0.117 | 0.113 | 0.910 | -0.048 | 0.117 | 0.146 | 0.945 |
| $\beta_{7}$ | 0.019 | 0.109 | 0.106 | 0.935 | 0.023 | 0.117 | 0.133 | 0.955 |
| $\beta_{8}$ | -0.012 | 0.117 | 0.111 | 0.940 | -0.011 | 0.120 | 0.144 | 0.960 |
| $\alpha_{1}$ | 0.024 | 0.069 | 0.068 | 0.940 | 0.027 | 0.069 | 0.070 | 0.945 |
| $\alpha_{2}$ | -0.028 | 0.063 | 0.068 | 0.945 | -0.032 | 0.065 | 0.069 | 0.945 |
| $\alpha_{3}$ | 0.014 | 0.130 | 0.139 | 0.960 | 0.017 | 0.132 | 0.142 | 0.955 |
|  | Nonlinear model with censoring rate 25% (100% converged) | | | | | | | |
| $\beta_{1}$ | -0.376 | 0.355 | 0.241 | 0.580 | -0.001 | 0.020 | 0.019 | 0.935 |
| $\beta_{2}$ | 0.375 | 0.318 | 0.244 | 0.625 | 0.001 | 0.019 | 0.018 | 0.930 |
| $\beta_{3}$ | -0.372 | 0.349 | 0.244 | 0.605 | 0.003 | 0.018 | 0.019 | 0.955 |
| $\beta_{4}$ | 0.380 | 0.340 | 0.243 | 0.605 | 0.002 | 0.018 | 0.018 | 0.945 |
| $\beta_{5}$ | -0.381 | 0.329 | 0.243 | 0.605 | -0.001 | 0.020 | 0.019 | 0.960 |
| $\beta_{6}$ | -0.368 | 0.385 | 0.274 | 0.620 | -0.002 | 0.023 | 0.023 | 0.940 |
| $\beta_{7}$ | 0.364 | 0.370 | 0.270 | 0.615 | 0.001 | 0.021 | 0.020 | 0.935 |
| $\beta_{8}$ | -0.394 | 0.378 | 0.273 | 0.630 | 0.001 | 0.025 | 0.023 | 0.915 |
| $\alpha_{1}$ | -0.370 | 0.422 | 0.469 | 0.890 | 0.042 | 0.463 | 0.465 | 0.930 |
| $\alpha_{2}$ | 0.370 | 0.473 | 0.469 | 0.880 | -0.028 | 0.513 | 0.465 | 0.905 |
| $\alpha_{3}$ | -0.215 | 0.113 | 0.108 | 0.485 | -0.010 | 0.109 | 0.109 | 0.940 |
|  | Nonlinear model with censoring rate 50% (100.0% converged) | | | | | | | |
| $\beta_{1}$ | -0.351 | 0.345 | 0.241 | 0.620 | 0.001 | 0.028 | 0.031 | 0.960 |
| $\beta_{2}$ | 0.320 | 0.337 | 0.239 | 0.680 | 0.007 | 0.025 | 0.029 | 0.970 |
| $\beta_{3}$ | -0.330 | 0.346 | 0.239 | 0.650 | -0.002 | 0.030 | 0.031 | 0.960 |
| $\beta_{4}$ | 0.336 | 0.345 | 0.243 | 0.670 | 0.000 | 0.031 | 0.030 | 0.925 |
| $\beta_{5}$ | -0.307 | 0.314 | 0.241 | 0.715 | -0.002 | 0.029 | 0.030 | 0.965 |
| $\beta_{6}$ | -0.310 | 0.406 | 0.275 | 0.675 | 0.002 | 0.038 | 0.038 | 0.935 |
| $\beta_{7}$ | 0.310 | 0.337 | 0.272 | 0.735 | 0.003 | 0.034 | 0.034 | 0.945 |
| $\beta_{8}$ | -0.352 | 0.385 | 0.275 | 0.680 | -0.002 | 0.040 | 0.038 | 0.935 |
| $\alpha_{1}$ | -0.212 | 0.530 | 0.582 | 0.950 | 0.108 | 0.538 | 0.586 | 0.970 |
| $\alpha_{2}$ | 0.258 | 0.587 | 0.577 | 0.935 | -0.065 | 0.538 | 0.581 | 0.980 |
| $\alpha_{3}$ | -0.121 | 0.138 | 0.133 | 0.825 | 0.071 | 0.140 | 0.137 | 0.925 |

**Web References**

1. Andersen, P.K. and R.D. Gill, *Cox's regression model for counting processes: a large sample study.* The annals of statistics, 1982: p. 1100-1120.

2. Prentice, R.L. and S.G. Self, *Asymptotic distribution theory for Cox-type regression models with general relative risk form.* The Annals of Statistics, 1983. **11**(3): p. 804-813.

3. Liu, L., *Semiparametric and nonparametric models for survival data.* 2004.

4. Huang, J.Z. and L. Liu, *Polynomial spline estimation and inference of proportional hazards regression models with flexible relative risk form.* Biometrics, 2006. **62**(3): p. 793-802.

5. Carroll, R.J., D. Ruppert, and L.A. Stefanski, *Measurement error in nonlinear models*. Vol. 105. 1995: CRC press.

6. Gray, R.J., *Spline-based tests in survival analysis.* Biometrics, 1994: p. 640-652.

7. Huber, P.J. *The behavior of maximum likelihood estimates under nonstandard conditions*. in *Proceedings of the fifth Berkeley symposium on mathematical statistics and probability*. 1967. Berkeley, CA: University of California Press.

8. Yu, Y. and D. Ruppert, *Penalized spline estimation for partially linear single-index models.* Journal of the American Statistical Association, 2002. **97**(460): p. 1042-1054.
